# Inflammation of the nasal mucosa is associated with susceptibility to experimental pneumococcal challenge in older adults

**DOI:** 10.1016/j.mucimm.2024.06.010

**Published:** 2024-10

**Authors:** Britta C. Urban, André N.A. Gonçalves, Dessi Loukov, Fernando M. Passos, Jesús Reiné, Patrícia Gonzalez-Dias, Carla Solórzano, Elena Mitsi, Elissavet Nikolaou, Daniel O’Connor, Andrea M. Collins, Hugh Adler, Andrew Pollard, Jamie Rylance, Stephen B. Gordon, Simon P. Jochems, Helder I. Nakaya, Daniela M. Ferreira

**Affiliations:** 1 Oxford Vaccine Group, Department of Paediatrics, University of Oxford, Oxford, UK; 2 Clinical Sciences, Liverpool School of Tropical Medicine, Liverpool, UK; 3 NIHR Oxford Biomedical Research Centre, Oxford, UK; 4 Infection, Immunity and Global Health, Murdoch Children’s Research Institute, Parkville, Victoria, Australia; 5 Department of Microbiology and Immunology at the Peter Doherty Institute for Infection and Immunity, The University of Melbourne, Parkville, Victoria, Australia; 6 University Hospital Aintree, Liverpool University Hospitals Trust, Liverpool, UK; 7 Malawi-Liverpool-Wellcome Clinical Research Programme, Blantyre, Malawi; 8 Leiden University Centre for Infectious Diseases, Leiden University Medical Centre, Leiden, The Netherlands; 9 Hospital Israelita Albert Einstein, São Paulo, Brazil; 10 Department of Clinical and Toxicological Analysis, School of Pharmaceutical Sciences, University of São Paulo, São Paulo, Brazil

## Abstract

*Streptococcus pneumoniae* colonization in the upper respiratory tract is linked to pneumococcal disease development, predominantly affecting young children and older adults. As the global population ages and comorbidities increase, there is a heightened concern about this infection. We investigated the immunological responses of older adults to pneumococcal-controlled human infection by analyzing the cellular composition and gene expression in the nasal mucosa. Our comparative analysis with data from a concurrent study in younger adults revealed distinct gene expression patterns in older individuals susceptible to colonization, highlighted by neutrophil activation and elevated levels of CXCL9 and CXCL10. Unlike younger adults challenged with pneumococcus, older adults did not show recruitment of monocytes into the nasal mucosa following nasal colonization. However, older adults who were protected from colonization showed increased degranulation of cluster of differentiation 8+ T cells, both before and after pneumococcal challenge. These findings suggest age-associated cellular changes, in particular enhanced mucosal inflammation, that may predispose older adults to pneumococcal colonization.

## INTRODUCTION

Older adults are at greater risk of disease from respiratory infection not only SARS-CoV-2 but also respiratory syncytial virus (RSV), influenza, *Haemophilus influenzae,* and *Streptococcus pneumoniae (pneumococcus)*[1], [2]. The mechanisms underlying this increased vulnerability are beginning to be unraveled and have been attributed to changes in the aging immune system. While aging is associated with many physiological and metabolic changes, it affects the immune system with increased immune-senescence and inflammaging, changes in the frequency and function of leukocytes, and alterations in the gut microbiome[3], [4], [5], [6]. An alternative and compatible view is that changes in immune cell composition and immune responses to recall antigens during aging are appropriate adaptations favoring recall responses rather than induction of primary responses upon exposure to pathogens. In this context, “inflammaging” is an early sign of underlying age-associated diseases and frailty whereas healthy aging is associated with balancing anti-inflammatory responses and longevity[7], [8].

Exposure to respiratory pathogens occurs in the upper respiratory tract and immunological responses here are critical for either the establishment or clearance of infection. Failure to clear infection increases the risk of microaspiration of pathogens into the lung and the development of pneumonia^9^. Since the emergence of SARS-CoV-2, there has been a renewed interest in mucosal immune responses in the upper respiratory tract during acute infection and convalescence with the aim to elucidate the immunological mechanisms that determine susceptibility to disease and pathogen transmission.

Community-acquired pneumonia affects young children (<5 years) and older adults disproportionately[10], [11], [12], [13], [14] and is a leading cause of death worldwide. *S. pneumoniae* is the primary cause of bacterial pneumonia and can reside in the upper respiratory tract without causing symptoms. Colonization with pneumococcus is a prerequisite of pneumococcal disease, with higher colonization levels increasing the risk of bacteria entering the lung^9^. Although older adults are at an increased risk of pneumococcal disease, the rates, duration, and densities of *S. pneumoniae* colonization in the upper respiratory tract are comparable or lower than those observed in younger adults[15], [16].

Controlled human infection models have the advantage that they allow the investigation of risk factors before infection as well as close monitoring of the kinetics of both systemic and mucosal immune responses during exposure to a pathogen. We recently reported the results of a controlled human infection challenge study where we exposed healthy older adults (over 50 years) to pneumococcus^15^. We observed in older adults that colonization rates and densities were not different from that of younger adults but their immune response differed – those who became colonized did not exhibit the expected boost in specific immunoglobulin G (IgG) against the inoculated strain capsular polysaccharide (CPS), as seen in younger adults^15^. In addition, in those participants who were challenged and did not become colonized, IgG levels to the CPS decreased a month after the challenge. A second experimental challenge of these study participants with homologous pneumococcus 6B up to a year later did not protect them from colonization, unlike in younger adults where re-challenge conferred protection[15], [17]. Together, these findings suggested a diminished immunizing effect of pneumococcal colonization in older adults, potentially increasing their vulnerability to severe pneumococcal disease even with lower colonization rates^18^.

To understand the differences in mucosal immune responses to exposure to pneumococcus between older and younger adults, we analyzed the cellular composition and transcriptome of nasal cells collected during this study and compared these with existing data from a concurrent experimental pneumococcal challenge models conducted in younger adults^19^.

Remarkably, we observed that older adults who became colonized presented with a susceptibility profile both at the transcriptional and cellular level before the pneumococcal challenge. Older but not younger adults susceptible to colonization displayed a distinct pattern of activated gene pathways associated with neutrophil degranulation and increased concentrations of CXCL9 and CXCL10 in the nasal mucosa at baseline. By contrast, protected older adults showed an increase in CD107a expression on a cytotoxic cluster of differentiation (CD)8+ T cells and upregulation of chemokines and cytokines within 2 days of exposure. Our results open the possibility of predicting susceptibility to pneumococcal colonization and greater risk of pneumococcal disease in older adults which would facilitate better prevention and treatment.

## RESULTS

### Distinct gene expression patterns in the nasal mucosa of older adults linked to carriage susceptibility

We previously conducted an experimental human pneumococcal challenge trial in healthy, older adults^15^ during which we collected nasal lining fluid and nasal microbiopsies to determine soluble immune mediators, nasal cell phenotype, and transcriptome from a subset of study participants. An overview of the sample schedule relative to inoculation with serotype 6B for older adults is provided in Supplementary Fig. 1. No demographic differences were seen between study participants who were susceptible to (*n* = 22 for cellular data, *n* = 17 for transcriptome data) or protected (*n* = 35 for cellular data, *n* = 24 for transcriptome data) from colonization (Table 1). Of note, we use the terms “protected” and “susceptible” study participant to allow a clear distinction between study participants at baseline (before pneumococcal challenge) who subsequently became carriage-positive or remained carriage-negative. Although we cannot completely rule out that a negative carriage status is a random event, our data here and from a previous study show that carriage-negative study participants mount an active immune response in the nasal mucosa within 24–48 hours of inoculation^20^.

**Table 1:** Demographic information.

Variables				All samples			RNA-Seq samples		
			Total (*n* = 57)	Protected (*n* = 35)	Susceptible (*n* = 22)	*p*	Protected (*n* = 24)	Susceptible (*n* = 17)	*p*
Sex, *n* (%)									
	Female		34 (60)	18 (51)	16 (73)	0.187	12 (50)	12 (71)	0.45
		50–64 y	16 (28)	7 (20)	9 (41)	0.252	4 (17)	6 (35)	0.428
		over 65 y	18 (32)	11 (31)	7 (32)		8 (33)	6 (35)	
Age, Mean ± SD			64.49 ± 7.53	65.23 ± 7.24	63.32 ± 8.01	0.368	66.04 ± 8.14	65 ± 7.3	0.138
Ex-smoker, *n* (%)									
	Yes		20 (35)	15 (43)	5 (23)	0.206	12 (50)	4 (24)	0.287
		50–64 y	7 (12)	5 (14)	2 (9)	0.504	2 (8)	1 (6)	0.123
		over 65 y	13 (23)	10 (29)	3 (14)		10 (42)	3 (18)	
HTN, *n* (%)									
	Yes		9 (16)	7 (20)	2 (9)	0.458	4 (17)	2 (12)	0.63
		50–64 y	2 (4)	2 (6)	0 (0)	0.75	0 (0)	0 (0)	0.434
		over 65 y	7 (12)	5 (14)	2 (9)		4 (17)	2 (12)	
Renal disease, *n* (%)									
	Yes		3 (5)	2 (6)	1 (5)	1	1 (4)	1 (6)	0.84
		50–64 y	1 (2)	1 (3)	0 (0)	0.935	0 (0)	0 (0)	0.482
		over 65 y	2 (4)	1 (3)	1 (5)		1 (4)	1 (6)	
Previous malignancy, *n* (%)									
	Yes		1 (2)	1 (3)	0 (0)	1	0 (0)	0 (0)	0.281
		over 65 y	1 (2)	1 (3)	0 (0)	1	0 (0)	0 (0)	0.129
Statin, *n* (%)									
	Yes		8 (14)	5 (14)	3 (14)	1	4 (17)	3 (18)	1
		50–64 y	1 (2)	1 (3)	0 (0)	0.956	0 (0)	0 (0)	0.517
		over 65 y	7 (12)	4 (11)	3 (14)		4 (17)	3 (18)	
Charlson index, *n* (%)									
	1		15 (26)	8 (23)	7 (32)	0.399	6 (25)	3 (18)	0.072
	2		27 (47)	15 (43)	12 (55)		9 (38)	12 (71)	
	3		13 (23)	10 (29)	3 (14)		8 (33)	2 (12)	
	4		2 (4)	2 (6)	0 (0)		1 (4)	0 (0)	
Charlson index ignoring age, *n* (%)									
	0		56 (98)	34 (97)	22 (100)	1	24 (100)	17 (100)	0.281
	2		1 (2)	1 (3)	0 (0)		0 (0)	0 (0)	

HTN = hypertension; RNA-seq = ribonucleic acid sequencing; SD = standard deviation.

**Fig. 1:**
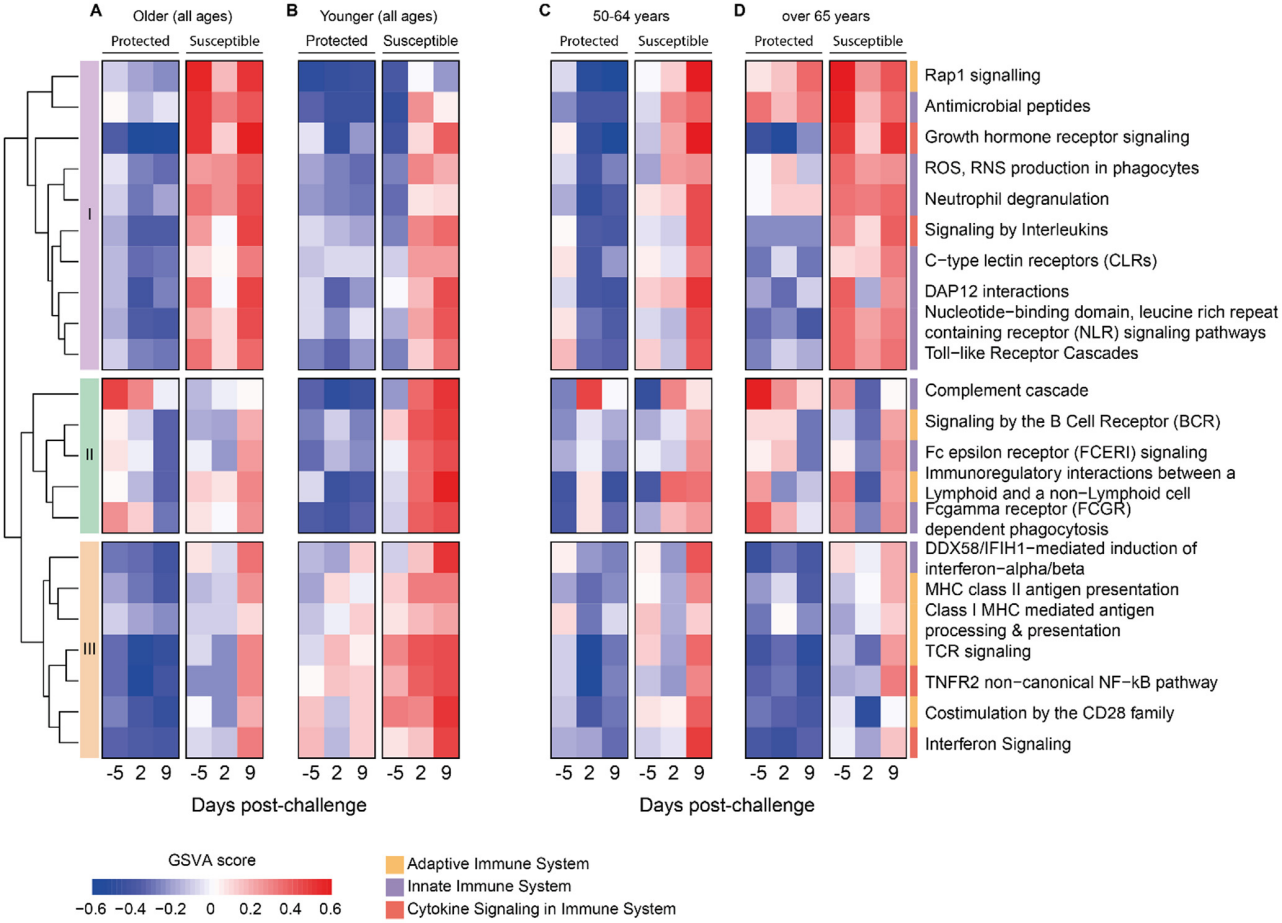
Gene expression analysis of nasal cells showed distinct patterns in older adults susceptible to carriage before pneumococcal challenge. (A) Gene set variation analysis (GSVA) of immune response pathways of nasal cells collected before (day −5) and after (day 2, day 9) pneumococcal challenge of older and (B) younger study participants who were protected (carriage-negative) or susceptible (carriage-positive), (C) older study participants between 50 to 64 years old who were protected (carriage-negative) or susceptible (carriage-positive), (D) older study participants over 65 years old who were protected (carriage-negative) or susceptible (carriage-positive). Based on Euclidean distance calculation, we identified three groups: group I contains gene sets associated predominantly with innate immune responses, group II and III are enriched for gene sets associated with antibody-mediated and adaptative immune responses, respectively. CD = cluster of differentiation; MHC = Major histocompatibility complex

We first investigated which immune response pathways were enriched in the nasal transcriptome of protected and susceptible older adults before (day −5) and after (day 2 and day 9) pneumococcal challenge using gene set variation analysis (GSVA) (Fig. 1A, Supplementary Data file 1). Based on Euclidean distance clustering, gene sets fell into three distinct groups with different enrichment patterns over time and carriage status. Group I gene sets were associated with innate immune responses and cytokine signaling including pathways such as “Antimicrobial peptides”, “Neutrophil degranulation”, “DAP12 interactions” and “Toll-like receptor cascades”. They remained higher across all time points in susceptible older adults but were not induced in protected older adults after the pneumococcal challenge. By contrast, group II gene sets were largely associated with antibody-mediated processes such as “Complement Cascade” and “Fc gamma receptor-dependent phagocytosis”. These pathways were predominantly enriched at baseline (day −5) in protected older adults and induced in susceptible older adults by day 9. Group III gene sets associated with antigen presentation and T cell activation and were induced in susceptible study participants on day 9.

We then asked whether similar gene pathway patterns were evident in younger adults before and after the pneumococcal challenge using a control group from our previously published studies (protected *n* = 6, susceptible *n* = 8, age <50 years)[19], [21]. The study in younger adults was independent of the study in older adults reported here but conducted in parallel at the same time and by the same team, using the same pneumococcal serotype 6B batch for inoculation and SOPs for inoculation and detection of bacteria. In younger adults, GSVA of the nasal cell transcriptome showed no difference in the expression of group I or group II gene sets between protected and susceptible study participants at baseline. However, gene pathways of both groups were rapidly induced after pneumococcal challenge in susceptible younger adults indicating activation of innate and antibody-mediated immune responses (Fig. 1B and Supplementary Data file 2).

To obtain a more granular picture of transcriptomic changes with age, we split older adults into two age groups (50–64 years *n* = 16, over 65 years *n* = 25) and repeated GSVA analysis for each group separately. Notably, in older adults between 50 and 64 years of age, group I genes were only moderately activated in susceptible study participants whereas susceptible older adults over the age of 65 years showed strong expression of all group I gene sets before the pneumococcal challenge (Figs. 1C and 1D, Supplementary Data files 3 and 4). In addition, protected adults over the age of 65 years but not those aged between 50 and 64 years showed high expression levels of gene sets associated with “Complement Cascade”, "Immunoregulatory interactions between a Lymphoid and non-Lymphoid cell” and “Fc gamma receptor-dependent phagocytosis” at baseline (Fig. 1D).

In summary, gene expression patterns in the nasal mucosa were fundamentally different between age groups. Susceptible older adults exhibited a unique gene set signature associated with an activated innate immune response profile before the pneumococcal challenge which was more pronounced in older adults over the age of 65 years.

### Co-expression analysis of genes reveals upregulation of neutrophil degranulation and inflammatory pathways in the nasal mucosa of susceptible older adults

**Fig. 2:**
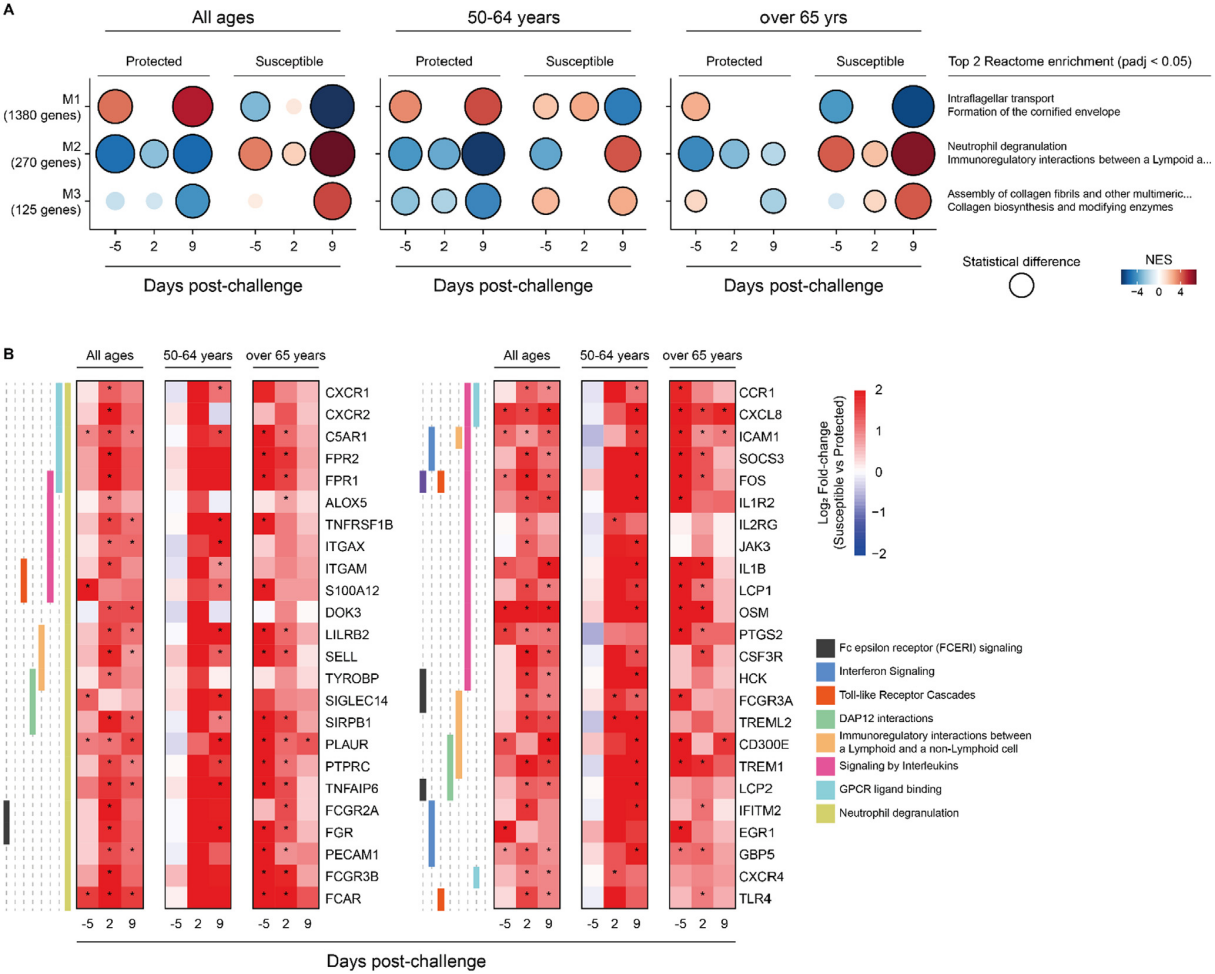
Co-expression analysis of nasal cell transcripts by carriage status before and after pneumococcal challenge. (A) Co-expressed genes were determined by CEMiTool (Co-Expression Module identification Tool), assessing normalized gene expression profile by carriage status (susceptible and protected) before (day −5) and after (days 2 and 9) pneumococcal challenge. Over-Representation Analysis (enrichr function from R package clusterProfiler) was used to determine the biological function of each module genes using Reactome database (level 3 gene sets). Gene Set Enrichment Analysis (GSEA) tool and z-score values for each gene across all timepoints were used to determine whether module genes were positively (positive NES values) or negatively (negative NES values) enriched. Overexpression analysis is shown for all older adults (all ages), 50-64 years old and over 65 years old study participants. (B) Heatmap showing log2 fold-change of overexpressed genes within each pathway before and after pneumococcal challenge (susceptible vs protected samples) for all older adults (all ages), 50–64 years old and over 65 years old study participants. Criteria of differentially expressed genes were absolute log2 fold-change >1 and non-adjusted *p* value <0.05.

To develop a deeper understanding of susceptibility and protection from pneumococcal carriage in older adults we explored co-expressed genes and biological pathways associated with colonisation^22^. Co-expression analysis identified three modules (M1–M3; Fig. 2A) with module M1 genes enriched in protected and M2 genes enriched in susceptible older adults at baseline (Supplementary Fig. 2A). Notably, genes within the M2 module were enriched for the reactome pathways “Neutrophil Degranulation”, “Immunoregulatory interactions between a Lymphoid and a Non-lymphoid Cell” and “Signalling by Interleukins” (Fig. 2A) in older adults over the age of 65 years in line with upregulated gene sets identified by GSVA.

Next, we asked which genes were overexpressed in susceptible compared with protected older adults at baseline in Module M2. Of the 270 co-expressed M2 genes, 216 were differentially expressed at at least one timepoint (Supplementary Data file 5). Network analysis of genes overexpressed in susceptible study participants before the challenge (Supplementary Fig. 2B) revealed a more granular picture of interlinked immune response pathways. Genes overexpressed within the neutrophil degranulation pathway at baseline in susceptible study participants over the age of 65 years (Fig. 2B) included *PLAUR* (2.9-fold, *p* = 1.3 × 10^−5^), encoding the Plasminogen Activator Urokinase receptor, which is upregulated by immune activation with the soluble form having been proposed as a marker for chronic inflammation^23^. *TREM1* (3.47-fold, *p* = 0.0003) signals through the DAP12 complex resulting in enhanced survival of immune cells and increased production of pro-inflammatory cytokines[24], [25] whereas *SIRPB1* (2.89-fold, *p* = 0.0001) results in activation of myeloid cells in conjunction with DAP12^26^. In a mouse model of pneumococcal pneumonia, TREM1 is critical for the migration of neutrophils to the lung and enhanced early response of alveolar macrophages^27^. Of the Fc receptors, *FCAR* encoding the FcαR (3.84-fold, *p* = 0.0078), *FCGR3A* (1.8-fold, *p* = 0.001) and *FCGR3B* (2.9-fold, *p* = 0.004) encoding the FcγRIIIa and FcγRIIIb, were overexpressed at baseline before challenge in susceptible study participants over the age of 65 years (Fig. 2B). Of the M2 cytokine and chemokine genes, *IL1B* (3.035-fold, *p* = 0.0005), *CXCL8* (3.47-fold, *p* = 2.5 × 10^−5^) and oncostatin M (*OSM*, 3.9-fold, *p* = 2.24 × 10^−5^) showed higher expression in nasal cells of susceptible compared with protected older adults over the age of 65 years at baseline. Oncostatin M drives bacterial-induced airway inflammation and asthma exacerbation and has also been reported to be increased in patients with severe community-acquired pneumonia[28], [29], [30]. Of the “Interferon Signaling” genes, *EGR1* encoding the early growth response 1 zinc-finger transcription factor involved in inflammatory responses and *GBP5* encoding the guanylate binding protein 5 which stimulates assembly of NLRP3 inflammasome were 2.4-fold (*p* = 0.0003) and 1.1-fold (*p* = 0.01) overexpressed in susceptible older adults over the age of 65 years (Supplementary Table 1).

Overall, the nasal transcriptome of susceptible older adults over the age of 65 years showed increased inflammation and neutrophil activation before challenge with pneumococcus.

### Increased baseline expression of CXCL9 and CXCL10 in the nasal mucosa of susceptible older adults

**Fig. 3:**
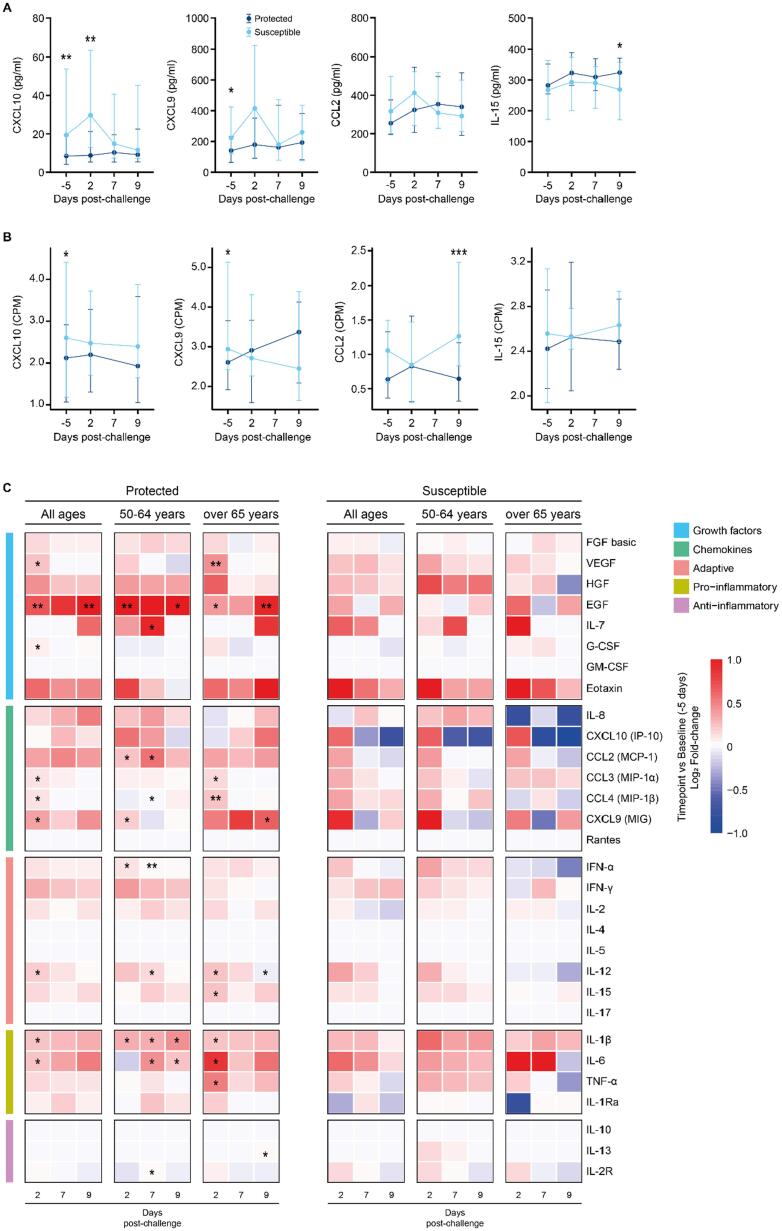
Changes in cytokine concentration in the nasal lining fluid of older adults experimentally challenged with S. pneumoniae 6B. (A) Line graphs showing the median and interquartile range of cytokine concentrations in the nasal lining fluid in older study participants before (day −5) and after (day 2, day 7, and day 9) pneumococcal challenge in susceptible (light blue line, *n* = 22) and protected (dark blue line, *n* = 35) study participants. In protected study participants, the concentration of IL-15 was higher at day 9 (Mann-Whitney U test, *p = 0.0344), and in susceptible study participants concentration of CXCL10 (IP-10) was higher at day −5 and day 2 (Mann-Whitney U test, *p = 0.00116, **p = 0.0044) and the concentration of CXCL9 (MIG) was higher at day −5 (Mann-Whitney U test, *p = 0.0419). No differences were observed for CCL2 (MCP-1). (B) Line graphs showing the median and interquartile range of cytokine normalized gene expression (CPM = counts per million) in older study participants before (day −5) and after (day 2, and day 9) pneumococcal challenge in susceptible (light blue line, *n* = 18) and protected (dark blue line, *n* = 24) study participants. In susceptible study participants normalized gene expression of CXCL10 (IP-10) and CXCL9 (MIG) was higher at day −5 (DESeq2 Wald test, *p = 0.00303, *p = 0.0210, respectively), and CCL2 was higher at day 9 (DESeq2 Wald test, ***p = 0.000218). No differences were observed for IL-15. (C) Heatmap depicting log2 fold-change at day 2, 7, and 9 relative to baseline (day −5) samples for all older adults (all ages), 50–64 years old and over 65 years old study participants. The cytokines were grouped based on function: adaptive, anti-inflammatory, chemokines, growth factors, and pro-inflammatory responses. The statistical test performed was the non-parametric Wilcoxon test. One asterisk (*) indicates *p* value <0.05, and two asterisk (**) for *p* value <0.01. IFN = interferon; IL = Interleukins.

Given the increased expression of genes associated with inflammation and neutrophil activation in susceptible older adults, we asked whether transcriptional changes are at least in part reflected at the protein level in the nasal mucosa (Fig. 3 and Supplementary Figs. 3 and 4). The median concentration of the chemokine CXCL10 (IP10) was 2.27-fold higher before (day −5) and 3.4-fold higher on day 2 after the challenge in the nasal lining fluid of susceptible compared with protected study participants. Likewise, the concentration of the related chemokine CXCL9 was 1.16-fold increased at baseline (day −5) in susceptible older adults (Fig. 3A). Expression of the genes encoding these two chemokines was also increased in susceptible compared with protected older adults at baseline only (Fig. 3B, *CXCL9* 2.1-fold, *p* = 0.021; *CXCL10* 2.6-fold, *p* = 0.003). When considering these changes in 50–64 years and over 65 years old study participants, higher levels of CXCL10 (3.3-fold, *p* = 0.018) at baseline were observed in susceptible compared to protected older adults over the age of 65 years only (Supplementary Fig. 3A). Although expression of CXCL9 and CXCL10 is induced by interferon (IFN)γ, IFNγ concentration in nasal lining fluid did not differ between susceptible and protected older adults (Supplementary Fig. 3B). However, we observed a positive correlation between IFNγ and CXCL9 and CXCL10, respectively, in 50–64 years and over 65 years old study participants (Supplementary Fig. 3C). These data are reminiscent of younger adults with an asymptomatic viral infection or receiving Live Attenuated Influenza Vaccine, who showed high baseline levels of CXCL10 and increased susceptibility to pneumococcal colonization upon challenge^19^. As with younger adults, there were no differences between CCL2 concentrations between susceptible and protected older adults. Unlike in younger adults, we did not observe an association between CCL2 concentration and the frequency of monocytes in the nasal mucosa (Supplementary Fig. 4A). However, *CCL2* gene expression was four times higher in susceptible compared with protected older adults at day 9 after challenge (*p* = 0.001; Fig. 3B). In addition, the concentration of interleukin (IL)-15, a cytokine which regulates T cell proliferation and activation, gradually increased in protected study participants after challenge and was 1.3-fold higher at day 9 compared with susceptible study participants. There were no statistically significant differences between protected and susceptible study participants before and after the pneumococcal challenge for all other cytokines analyzed (Supplementary Fig. 4B).

When considering induction of cytokines for susceptible or protected study participants (log_2_ fold-change for day 2, 7, and 9 against baseline) across all ages, or split into 50–64 and over 65 years of age, we observed responses to pneumococcal challenge in the nasal mucosa of protected but not susceptible study participants (Fig. 3C and Supplementary Data file 6). Epidermal Growth Factor (EGF) significantly increased from baseline in protected study participants on days 2 and 9 and whereas Vascular Endothelial Growth Factor (VEGF) increased on day 2 in the over 65 years old only. In protected study participants between 50 and 64 years old, the concentration of CCL2, CXCL9, IFNα, and IL1β increased from baseline to day 2 whereas in over 65 years old, the concentrations of MIP1α, MIP1β, IL12, IL15, Il1β, IL6 and tumor necrosis factor α increased from baseline to day 2. This is in contrast to observations in protected younger adults[19], [20] who showed rapid induction of nasal cytokines which peaked within 24 hours after challenge but were undetectable by 48 hours (day 2) indicating that protected older adults may show a slight delay in nasal cytokine responses to pneumococcal challenge. Cytokine levels at baseline did not correlate with age in study participants (Supplementary Fig. 4B).

### Lack of monocyte recruitment in susceptible older adults after pneumococcal challenge

The nasal cell gene expression profiles suggested that older adults susceptible to pneumococcal colonization had higher levels of innate immune cell activation compared with protected older adults. Granulocytes are abundant in the nasal mucosa while monocytes are sparse with the latter rapidly recruited after challenge in younger adults[19], [31].

**Fig. 4:**
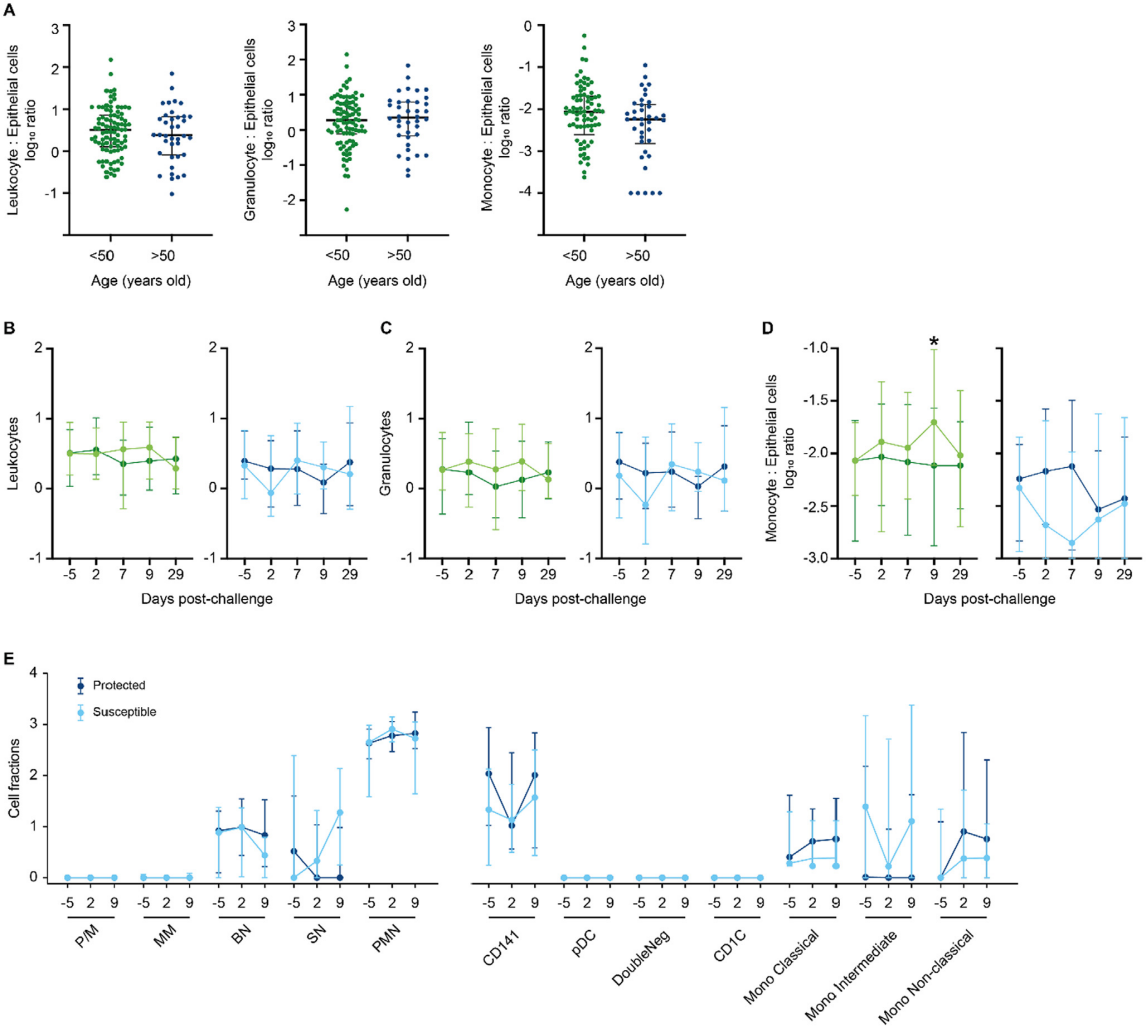
Recruitment of monocytes into the nasal mucosa of susceptible older adults is impaired. (A), In nasal micro-biopsies, the number of leukocytes, granulocytes and monocytes was comparable between younger (green, *n* = 91) and older adults (blue, *n* = 38) before pneumococcal challenge (day −5). Cell numbers of each leukocyte subset were normalized to epithelial cells as a ratio to account for differences in the total number of cells obtained for each biopsy. (B–D), Line graphs showing median and interquartile range of leukocytes, (B), granulocytes (C), and monocytes (D) in younger and older study participants before (day −5) and after (day 2, day 7, day 9, and day 29) pneumococcal challenge in susceptible (young: light green *n* = 28; old: light blue line, *n* = 22) and protected (young: dark green, *n* = 39; old: dark blue line, *n* = 35) study participants. The proportion of monocytes increased at day 9 after challenge in susceptible compared to protected younger adults (Mann Whitney U test, *p = 0.0122). This recruitment of monocytes into the nasal mucosa was not observed in susceptible older adults (*n* = 15). (E), Deconvolution analysis of granulocytes and myeloid cells using CIBERSORTx. Normalized gene expression log2 CPM (Counts Per Million) and granulocytes and monocytes databases were used to determine the estimated cell population proportion. BN = band neutrophils; CD = cluster of differentiation; CD141 = CD141+ dendritic cells; CD1c-CD141-dendritic cells = double negative; CD1C = CD1c+ dendritic cells; MM = metamyelocytes; mono = monocytes; pDC = plasmacytoid dendritic cells; PMN = polymorphonuclear; P/M = promyelocytes/myelocytes; SN = segmented neutrophils.

We analyzed the cellular composition of nasal cells by flow cytometry from nasal microbiopsies which were obtained in parallel to those used for transcriptome analysis. Where comparable data were available from our control group of younger adults from an independent study conducted in parallel, we contrasted data from younger and older adults. The overall number of leukocytes, granulocytes, and monocytes as a ratio to epithelial cells[19], [32] were comparable between younger and older adults before the pneumococcal challenge (Fig. 4A). After the challenge, there were no differences in the proportion of leukocytes or granulocytes over the following 29 days between susceptible and protected study participants in either younger or older adults (Figs. 4B and 4C). In younger adults, recruitment of monocytes peaked at day 9 after pneumococcal challenge in susceptible study participants and was significantly different from baseline (median, 25th and 75th percentile: day −5, 0.0086, 0.004–0.019; day 9, 0.019, 0.007–0.09; *p* = 0.0012 Wilcoxon matched-pairs signed rank test). In contrast to younger adults^19^, monocytes were not recruited into the nasal mucosa of susceptible older adults in response to the pneumococcal challenge (Fig. 4D). Although there was a trend toward a drop in monocyte frequencies in susceptible older adults after the challenge, variability within the data was high. When we split data from older study participants into 50–64 years and over 65 years old study participants, no further differences between these age groups became apparent (Supplementary Fig. 5A).

Our cellular data did not allow us to delineate innate cell subsets beyond the limited number of surface markers we had available for granulocytes or due to the low cell count for monocytes (Supplementary Fig. 5B). We, therefore, applied deconvolution analysis of mixed cell populations obtained in our bulk transcriptome data. Based on this analysis, mature neutrophils were the most abundant granulocyte population in the nasal mucosa independent of carriage status and timepoint (Fig. 4E). Myeloid cells were more heterogenous with CD141+ cDC1 the most abundant population in both susceptible and protected study participants. Intermediate monocytes appeared more abundant in susceptible participants at baseline although these differences were not statistically significant. Intermediate monocytes display an activated phenotype and are generally associated with more inflammatory conditions^33^. Notably, they were enriched in the nasal mucosa of older adults but not younger adults infected with influenza A virus, and their frequency correlated with age^31^.

### Activation of neutrophils is associated with susceptibility to carriage in older adults

**Fig. 5:**
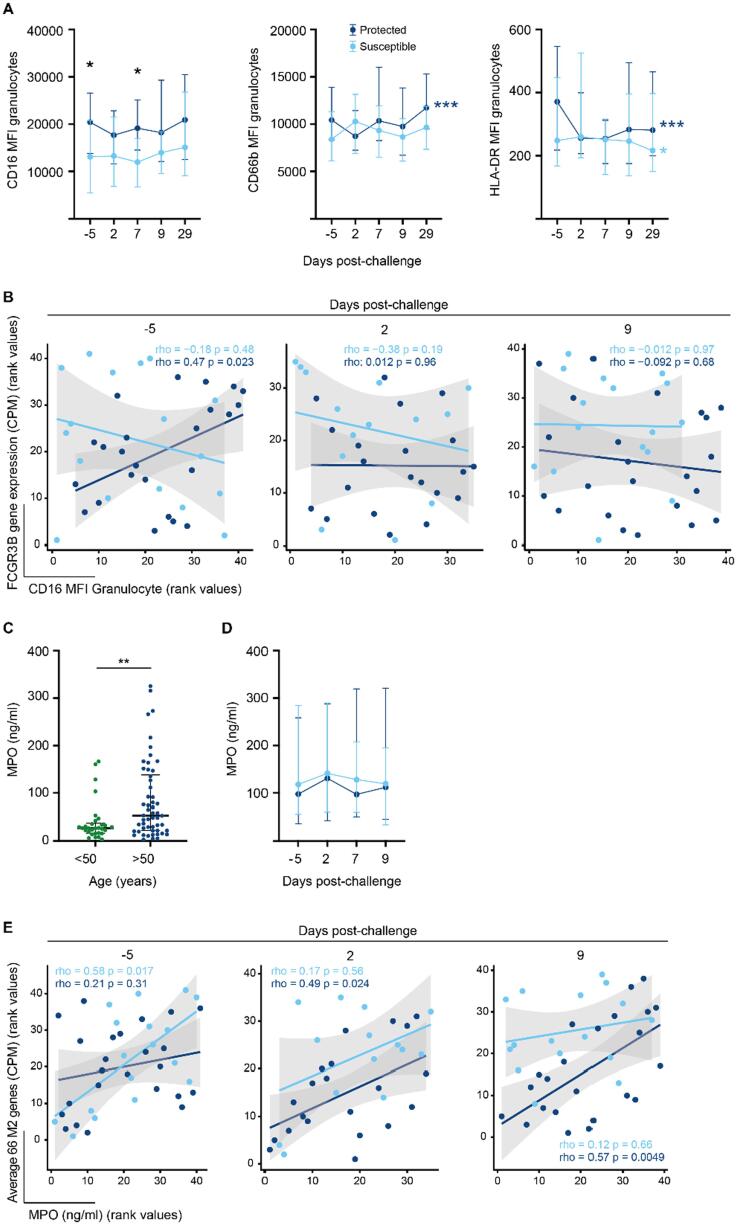
Granulocytes are activated in older adults susceptible to pneumococcal colonization. (A), Line graph (median and interquartile range) of expression levels of CD16, CD66b and HLA-DR on granulocytes before (day −5) and after challenge (day 2, day 7, day 9, and day 29) in protected (dark blue, *n* = 34) and susceptible (light blue, *n* = 22) older adults. The expression level of CD16 was significantly lower on nasal granulocytes at baseline and day 7 (Mann Whitney U test, baseline: *p* = 0.034, day 7: *p* = 0.014). Expression levels of CD66b changed significantly over time in protected older study participants (Friedman statistic 22.67, *p* = 0.0001) and for HLA DR in both protected (Friedman statistic 19.14, *p* = 0.0007) and susceptible (Friedman statistic 9.7, *p* = 0.044) study participants. (B), Correlation between expression levels of FCGR3B gene expression and CD16 on the surface of granulocytes at baseline (day −5), day 2 and day 9 in protected and susceptible study participants. (C), At baseline (day −5), the concentration of MPO (Myeloperoxidase) in nasal lining fluid in older adults (*n* = 54) was higher than in younger adults (*n* = 30). Shown are dot plots with median and interquartile range, Mann Whitney U test **p = 0.0085. (D), The concentration of MPO did not change after pneumococcal challenge in older study participants who became susceptible (light blue, *n* = 22) or remained protected (dark blue, *n* = 33). Line graph showing median and interquartile range of MPO concentration before and after inoculation with pneumococcus. (E), Correlation between expression levels of 66 M2 module genes associated with the Reactome pathway “neutrophil degranulation” and MPO concentration in nasal lining fluid at baseline (day −5), day 2 and day 9 in protected and susceptible study participants. CD = cluster of differentiation.

Since susceptible older adults showed enrichment of gene pathways associated with neutrophil degranulation before the challenge (Fig. 1, Fig. 2), we investigated the activation of granulocytes and monocytes before (day −5) and after (days 2, 7, 9, 29) pneumococcal challenge. The expression level of CD16—but not CD66b or HLA DR—was consistently lower on granulocytes of older adults susceptible to pneumococcal colonization with significant lower levels observed at baseline and day 7 after challenge (Fig. 5A and Supplementary Fig. 6A). However, after analysis of expression levels of CD16, CD66b and HLA DR on granulocytes in 50–64 years and over 65 years older adults separately, these differences were no longer apparent (Supplementary Fig. 6B). Furthermore, within each group of protected and susceptible study participants expression of CD66b and HLA DR changed after pneumococcal challenge in protected study participants and for HLA DR in susceptible study participants (Fig. 5A)[34], [35] Higher expression of CD16 on granulocytes positively correlated with gene expression levels for *FCGR3B* in protected but not susceptible older adults at baseline (Fig. 5B). Since CD16 expression on neutrophils and phagocytosis of bacteria is reduced in older adults^36^, our data suggest that granulocytes from older adults susceptible to pneumococcal carriage have reduced phagocytic capacity despite higher gene expression levels. Alternatively, cleavage of CD16 from the surface of granulocytes could indicate that these cells may have been already activated before challenge in older adults susceptible to pneumococcal carriage.

Opsonization, degranulation, and radical oxygen species production are important defense mechanisms against colonization with pneumococcus^37^. To determine whether neutrophil degranulation increased after challenge with pneumococcus as we reported for younger adults^19^, we measured the concentration of myeloperoxidase (MPO) in nasal lining fluids. MPO is stored in azurophil neutrophil granules which, upon activation of neutrophils, can fuse with phagolysosomes or the plasma membrane to release their content. MPO in extracellular spaces is therefore a marker of recent neutrophil degranulation and has been associated with local tissue damage. Before challenge, the concentration of MPO in the nasal lining fluid was 2-fold higher in older adults compared with younger adults (Fig. 5C). Unlike carriage-positive younger adults, levels of MPO in the nasal lining fluid did not increase after challenge in susceptible older adults (Fig. 5D) across all ages or when analyzed separately for 50–64 years old and over 65 years old study participants (Supplementary Fig. 6C).

Next, we asked whether expression levels of the 66 genes associated with the neutrophil degranulation pathway within the M2 module correlated with the MPO concentration in nasal lining fluid (Fig. 5E). MPO levels positively correlated with expression levels of neutrophil degranulation M2 module genes in susceptible older adults before but not after challenge suggesting that granulocytes were refractory to further activation. By contrast, in older adults protected from carriage, neutrophil degranulation genes within the M2 module correlated with MPO levels after but not before the challenge implying that granulocytes became activated and may have contributed to the clearance of pneumococci from the nose as we reported previously^20^. Together these data indicate that neutrophil degranulation occurred in susceptible older adults before the challenge with limited responses to pneumococci after the challenge.

### Activation of monocytes is associated with susceptibility to carriage in older adults

**Fig. 6:**
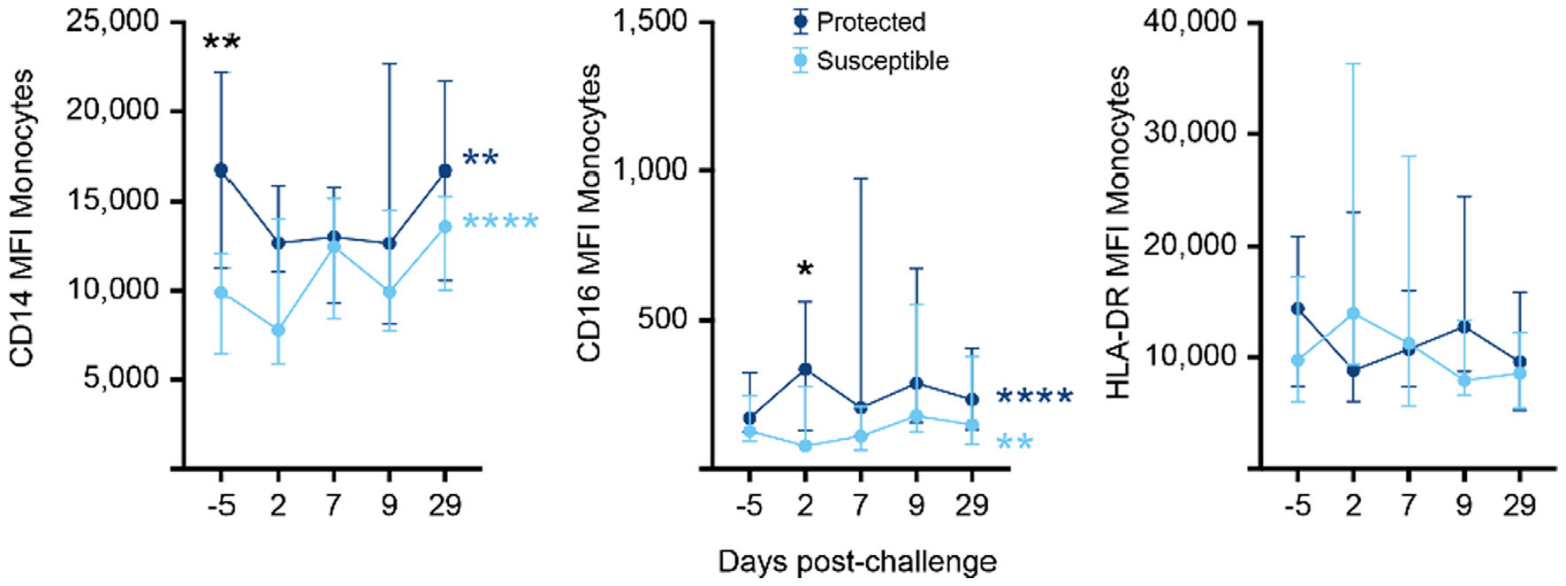
Expression of CD14 and CD16 is increased on monocytes of protected study participants. Line graph (median and interquartile range) of expression levels of CD14, CD16, and Human Leukocyte Antigen DR (HLA-DR) on monocytes before (day −5) and after challenge (day 2, day 7, day 9, and day 29) in protected (dark blue, *n* = 25) and susceptible (light blue, *n* = 14) older adults. The expression level of CD14 was significantly lower on nasal monocytes at baseline (Mann Whitney U test, baseline: *p* = 0.0019). The expression levels of CD14 and CD16 changed over time in both protected (CD14: Friedman statistic 18.01 *p* = 0.0012; CD16: Friedman statistic 27.98 *p* < 0.0001) and susceptible study participants (CD14: Friedman statistic 29.64, *p* < 0.0001; CD16 Friedman statistic 18.44, *p* = 0.001). CD = cluster of differentiation.

Given the lack of monocyte recruitment, we analyzed expression levels of CD14, CD16 and HLA DR on monocytes (Supplementary Fig. 7A). Expression levels of CD14 but not HLA DR or CD16 were significantly lower on monocytes before challenge (day −5) in susceptible compared with protected study participants (Fig. 6) indicating a dominance of intermediate monocytes in the nasal mucosa of susceptible older adults in agreement with deconvolution analysis of nasal transcriptome data (Fig. 4E). In protected older adults, the expression levels of CD16 increased on day 2 after challenge (Fig. 6). The expression levels of CD14 and CD16 on monocytes changed significantly in both protected and susceptible study participants after pneumococcal challenge (Fig. 6). When we analyzed data by age in 50–64 years old and over 65 years old study participants, increased CD14 expression on monocytes was evident in protected older adults at baseline in 50–64 years old study participants (Supplementary Fig. 7B; *p* = 0.02) but not those over the age of 65 years (*p* = 0.055). Within protected and susceptible older study participants, expression levels changed significantly for CD14 and CD16 but not for HLA DR relative to baseline (Fig. 6). Taken together these data indicate the absence or at least delay of an important clearance mechanism of pneumococci in the nasal mucosa that has been observed in younger adults^19^.

#### In older adults, T cell subsets in the nasal mucosa are reduced

**Fig. 7:**
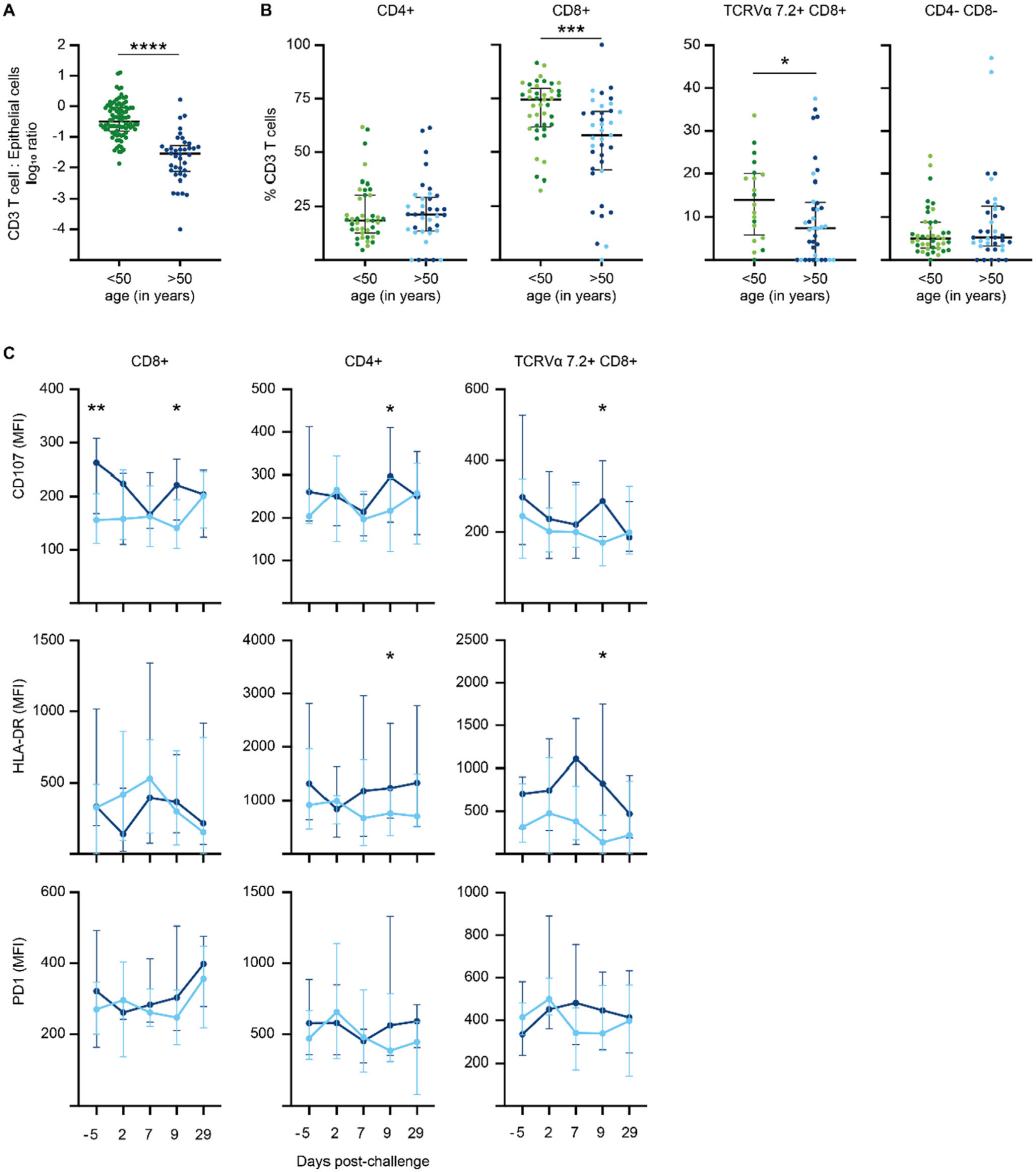
T cells are reduced in the nasal mucosa of older adults compared to younger adults. (A), In nasal microbiopsies, the number of CD3+ T cell subsets (as a log10 ratio with endothelial cells) is greatly reduced in older adults (blue) compared to younger adults (green) before pneumococcal challenge (day −5). (B), CD8+ and TCRVα7.2 CD8+ T cells are significantly lower in older compared to younger adults before pneumococcal challenge. Shown are dot blots of CD4+ T cells (younger adults *n* = 43, older adults *n* = 38), CD8+ T cells, TCRVα7.2 cells and DN T cells as percentage of CD3+ T cells, Mann Whitney U test, ***p < 0.001, ****p < 0.0001; (C), Expression levels of CD107a, PD1 and HLA DR on CD8+, CD4+ and TCRVα7.2 CD8+ T cells before and after challenge with Spn6B in protected (dark blue) and susceptible (light blue) older study participants. Line graphs show median and interquartile range at day −5 before and day 2, day 7 day 9 and day 29 after pneumococcal challenge. Mann Whitney U test *p < 0.05, **p < 0.01. CD = cluster of differentiation.

Finally, we analyzed the distribution and phenotypes of T cell subsets in protected and susceptible older study participants before and after the challenge with pneumococcus. The proportion of T cell subsets (as a ratio to epithelial cells) before the challenge (day −5) revealed that the frequency of total CD3+ T cells was 13.7-fold reduced in the nasal mucosa of older study participants compared with younger study participants (Fig. 7A; median (25th and 75th quartile) of CD3+T cell:epithelial cell ratio: 0.028 (0.008–0.058) for older adults; 0.384 (0.16–0.89) for younger adults, *p* < 0.0001 Mann-Whitney U test). The differences in total T cell frequency before challenge (day −5) were driven by a significantly smaller percentage of CD8+ T cells (Fig. 7B; median (25th and 75th quartile) CD8+ T cells: 57.7% (41.6%–68.9%) for older adults; 74.5% (61.6%–79.7%) for younger adults, *p* = 0.0004 Mann-Whitney U test) and TCRVα7.2+ CD8+ T cells—presumably mucosal-associated invariant T cells (MAIT) cells—between younger and older study participants [Fig. 7B; median (25th and 75th quartile) TCRaV7.2+ T cells: 7.4% (0%–13.5%) for older adults; 14% (5.9%–20%) for younger adults, *p* = 0.04 Mann-Whitney U test], but not double negative CD3+CD4-CD8- T cells.

Subsequently, we considered whether there were differences in the recruitment of T cell subsets in the nasal mucosa between susceptible and protected older and younger adults. Susceptible older adults showed a tendency toward lower percentage of CD4+ T cells than protected older study participants before (day −5) pneumococcal challenge but the difference was not significant [Supplementary Fig. 8A, median (25th and 75th quartile) CD4+ T cells: 23.5% (15.6%–34.2%) for protected older adults; 16.5% (12.6%–25.1%) for susceptible older adults, *p* = 0.06 Mann-Whitney U test].

In older study participants, we evaluated changes in expression levels of CD107a, PD1, and HLA DR on T cell subsets over time. CD8 T cells in the nasal mucosa of protected older adults showed higher expression levels of CD107a on CD8+ T cells at baseline compared with susceptible study participants [Fig. 7C; median and interquartile range CD107 MFI day −5: protected older adults 262 (168–308), susceptible older adults 156 (112–205), *p* = 0.0049 Mann-Whitney U test] which was more pronounced in study participants over the age of 65 years (Supplementary Fig. 8B).

In addition, CD8+, CD4+, and TCRVα7.2+ CD8+ T cells showed increased expression of CD107a 9 days after challenge in protected compared with susceptible study participants. However, when we split study participants into two age groups, increased expression of CD107a on day 9 was evident only in CD4+ T cells in study participants over the age of 65 years (Supplementary Fig. 8B). Expression levels of HLA DR increased on CD4 and TCRVα7.2+ CD8+ T cells on day 9 after challenge in protected but not susceptible study participants while there were no differences in expression levels of PD1 (Fig. 7C). Together these data suggest that cytotoxic CD8+ T cells in the nasal mucosa of older study participants—while reduced in frequency—may be associated with protection from carriage.

## DISCUSSION

Aging alters the innate immune response with changes in neutrophil recruitment into tissue, reduction in phagocytosis, killing of bacteria and antigen presentation by myeloid cells^38^ and the adaptive immune response with an increase in cytotoxic CD4+ and CD8+ T cells and an increase in age-associated B cells in the peripheral circulation[38], [39], [40], [41]. Here we investigated the immune response in nasal tissue before and after experimental human pneumococcal challenge in healthy older adults over the age of 50 years and compared results with existing data of younger adults^19^. Remarkably, we observed that older adults who became colonized presented with a susceptibility profile both at the transcriptional and cellular level before pneumococcal challenge with these changes more pronounced in older adults over the age of 65 years.

Analysis of the nasal transcriptome revealed overexpression of genes associated with the reactome pathway “neutrophil degranulation”, an increase in neutrophil activation and inflammatory cytokine secretion in older susceptible adults over the age of 65 years compared with those between 50 and 64 years old. Granulocytes, the majority of which are neutrophils, are abundant in the nasal mucosa and the first line of defense against bacterial infection through mechanisms such as antibody-mediated phagocytosis, production of radical oxygen species, degranulation, and Neutrophil Extracellular Trap (NETosis). While the frequency of granulocytes remained unchanged before and after pneumococcal challenge in older study participants, expression levels of genes associated with the reactome pathway “neutrophil degranulation” positively correlated with MPO concentration in nasal lining fluid indicating that recent degranulation of neutrophils associated with susceptibility to carriage. Although the exact driver of the increased activation of neutrophils we report here requires further investigation, the release of MPO may result in local tissue damage which in turn may increase adhesion and micro-invasion of pneumococci in the nasal mucosa^42^. In this context, it is noteworthy that the *FCAR* gene encoding the FcαR was 3-fold overexpressed in susceptible adults before the pneumococcal challenge. Although anti-pneumococcal secretory IgA1 is cleaved by the *S. pneumoniae* IgA1 protease (ZmpA), it is an important opsonin for other viral and bacterial pathogens.

In our study, the concentration of both CXCL10 and the related cytokine CXCL9 was increased at the protein level and transcriptional level in older susceptible compared with protected older adults before the pneumococcal challenge. CXCL10 and the closely related CXCL9 are largely induced by IFNγ and chemoattractants for natural killer- and T cells, important for the clearance of viral infections. Increased concentrations of CXCL10 have been reported in nasal lining fluid before challenge with RSV in those who developed cold symptoms^43^ and were associated with increased pneumococcal densities in the nasopharynx of younger study participants receiving the nasal Live Attenuated Influenza Vaccine or with asymptomatic viral infection before pneumococcal challenge^19^. In a recent study, high levels of CXCL10 in nasal lining fluids served as a marker for undiagnosed viral infections, with a distinct profile of high levels of CXCL10 together with neutrophilic inflammation in bacterial and viral co-infections^44^. However, it is unlikely that the increased CXCL10 concentration in our study was due to viral infection because individuals with a viral infection were excluded from our analysis. The role of CXCL10 in bacterial infections is less clear. Increased CXCL10 concentrations were associated with pneumonia in animal models of pneumococcal disease and were reported as a promising marker that distinguished between CAP caused by *Staphylococcus aureus* or *S. pneumoniae* with higher levels detected in pneumococcal CAP^45^. Furthermore, CXCL10 can be produced by tissue-infiltrating neutrophils which express its receptor CXCR3 and act on these neutrophils in an autocrine fashion followed by degranulation, Reactive Oxygen Species (ROS) production, and chemotaxis thus contributing to airway inflammation^46^.

High serum concentrations of CXCL9 are a strong predictor of cellular aging and cardiovascular health even in healthy adults^47^. It is produced by endothelial cells, macrophages, and neutrophils^48^. Whether CXCL9 is indicative of neutrophilic inflammation or produced by endothelial cells in the nasal mucosa in our study remains unclear but given its role in biological aging, its association with increased susceptibility to colonization with *S. pneumoniae* requires further investigation and if confirmed may offer an opportunity to identify older adults at greater risk of pneumococcal infection.

The mechanisms underlying neutrophilic inflammation in susceptible older adults before challenge with pneumococcus are not known. In younger adults, pre-existing neutrophil activation in the upper respiratory tract was associated with increased symptomatic disease in response to RSV infection^43^. In the gut, reciprocal interaction between neutrophils and the gut microbiome is well-established with dysregulation of innate responses associated with chronic inflammatory conditions^49^. Less is known about the microbiome of the upper respiratory tract. However, studies in both mice and humans have shown an association of *S. pneumoniae* colonization with changes in the microbiome in older individuals[50], [51]. Analysis of the microbiome and its association with susceptibility to pneumococcal carriage in older adults will require further investigation in future studies.

Another important observation was the lack of recruitment of monocytes into the nasal mucosa after pneumococcal challenge, unlike our previous observation in younger adults where activation of neutrophils and a likely CCL2-mediated influx of monocytes resulted in bacterial clearance^19^. In a mouse model of pneumococcal pneumonia, both CCL2 and CCR2 expression were crucial for additional recruitment and clearance of pneumococcus by monocytes^52^ and a recent study suggested that age is associated with higher expression of CCR2 on monocytes but reduced phagocytosis of *S. pneumoniae*^53^. It will be important to analyze the phenotype and function of monocytes over the course of colonization in a human experimental infection model in more detail, together with the frequency and function of dendritic cells, in healthy younger and older adults since both populations play a role in the activation of adaptive immune responses in the nasal mucosa and systemically.

CD107a is a marker for degranulation and is rapidly recycled. A recent study showed that expression of CD107a on CD8+ T cells is higher in the lung than in peripheral circulation presumably because T cells in the respiratory mucosa are constantly challenged by inhaled antigens^54^. In protected older adults, we observed an increase of CD107a on CD8+ T cells in the nasal mucosa before pneumococcal challenge. CD8+ T cells are more abundant than CD4+ T cells in the nasal mucosa^55^. CD107a expression levels, in parallel with HLA DR, were also increased on CD4+ T cells and TCRVα7.2+CD8+ T cells 9 days after challenge. We have previously shown that younger adults have more MAIT in their nasal mucosa and in the peripheral circulation exhibit elevated tumor necrosis factor α and IFNγ levels when exposed to pneumococcus^55^. MAIT cells are often defined by co-expression of TCRVα7.2 and CD161 or can be identified using MR1/5-OP-RU tetramer staining but expression of CD161 on MAIT cells and binding to MR1/5-OP-RU tetramer in the respiratory tract is more variable^56^. Since we stained nasal cells only for the TCRVα7.2 chain and not CD161, we cannot be certain that all of the detected TCRVα7.2+ CD8+ T cells were MAIT cells. A shift toward a higher proportion of cytotoxic CD8 and CD4 T cells together with an increase in memory B cells is associated with an aging immune system but more pronounced in centenarians which suggests that during healthy aging an experienced immune system maintains effective adaptive immune responses[41], [57].

Our study has several limitations: We analyzed the cellular composition and nasal gene expression in parallel and therefore missed the opportunity to test targeted hypotheses based on gene expression patterns in nasal cells. Furthermore, small cell numbers recovered from nasal microbiopsies limited the number of markers and functional analyses we could employ. Finally, we had no access to immune cells in the lung and therefore cannot confirm whether similar changes in immune cell composition and function were present. In future studies, single-cell ribonucleic acid sequencing (RNA-seq) and high dimensional flow cytometry will assist more in-depth analysis of mucosal immune responses in the upper and lower respiratory tracts.

In conclusion, susceptible older adults show increased inflammation in the nasal mucosa at the cellular, protein, and transcriptional levels compared with protected older adults before the pneumococcal challenge with those changes more pronounced in over 65-year-old study participants. Increased inflammation was not evident in susceptible younger adults before pneumococcal challenge but once colonized, innate immune responses were induced faster. By contrast, protected older adults showed a higher level of activated, degranulated CD8+ T cells before challenge which may indicate an effective T cell response to antigenic stimulation due to inhaled and microbial antigens. Since biological and chronological aging differs, the heterogeneity in a healthy older population is in agreement with studies that showed chronic inflammation preceding symptoms associated with comorbidities and frailty^38^. Physiological changes in older adults such as reduced mucociliary clearance and increased expression of host receptors binding *S. pneumoniae* could lead to increased microaspiration into their lungs[1], [58], [59], [60], [61]. Microaspiration of pneumococcus into the lung may result in more pronounced inflammation with increased tissue damage if neutrophils are recruited or activated in a similar manner to those observed in the nasopharynx. Neutrophils in the lung are found in increased frequency in the lung and contribute to inflammation during bacterial and viral infections[62], [63], [64]. In addition, adults at risk of pneumonia have a delay in lung efferocytosis - the phagocytic clearance of apoptotic cells - following community-acquired pneumonia^65^. In a mouse model of pneumococcal and viral co-infection, older mice showed rapid activation of neutrophils in the lung in the absence of increased bacterial burden suggesting that dysregulation of immune responses is a major factor contributing to pneumonia^66^. Although the upper and lower respiratory tract differs in composition in both type and frequency of specialized epithelial cells and immune cells, there is sufficient overlap between compartments as assessed at the single-cell level[67], [68], [69]. The age-associated susceptibility pattern we observed here may explain the conundrum of a higher rate of pneumococcal disease in older adults despite lower carriage rates.

## METHODS

### Study design and demographics

Healthy adults aged 50–84 were inoculated with an estimated 80,000 colony-forming units per nostril of *Streptococcus pneumoniae* serotype 6B (strain BHN418^29^, GenBank accession number ASHP00000000.1) as previously described[17], [70]. Adults over the age of 65 years are eligible for vaccination with the pneumococcal vaccine PPV-23. To ensure that no inadvertent bias due to vaccination was introduced, the study included older adults between the ages of 50 and 64 years. As shown in Adler et al.^15^, vaccination had no effect on pneumococcal carriage rates or density and did not confer protection against colonization. Key eligibility criteria included capacity to give informed consent, no immunocompromised state or contact with susceptible individuals or children, no pneumococcal or influenza vaccine or infection in the last 2 years and not having taken part in Experimental Human Pneumococcal Challenge (EHPC) studies in the past 3 years. All volunteers gave written informed consent, and research was conducted in compliance with all relevant ethical regulations. Participants who carried non-experimental pneumococcal strains at baseline (day −5) or had a viral infection were excluded from all analyses.

Detailed demographic data of older adults >50 years can be found in Adler et al.^15^ (2021). For comparison, data from younger adults who were part of a control group of an influenza co-infection study were re-analyzed[19], [21]. The two studies were conducted in parallel using the same clinical and laboratory methods and study teams. However, experimental analysis was conducted independently. Some data on baseline (day −5) nasal cell populations before inoculation with serotype 6B were presented in a pre-print^71^ but the analysis of baseline nasal cell data presented here is sufficiently different and substantially extends the data presented in the preprint.

### Determination of carriage

Nasal washes were performed at day −5 and on days 2, 7, 9, 14, 22, and 29 post-challenge, and colonization determined by the presence of pneumococcus in nasal wash samples at any time point post-challenge up to and including day 29, detected using classical microbiology[17], [70].

### Nasal curettage

Nasal cells were collected on days −5, 2, 7, 9, and 29 by scraping the inferior turbinate from participants consecutively using four curettes (Rhino-pro, Arlington Scientific), as described previously^32^. Two curette samples were collected into Phosphate-Buffered Saline (PBS) + 2.5 mM Ethylenediaminetetraacetic Acid (EDTA) and 0.5% heat inactivated Fetal Bovine Serum (FBS) for immunophenotyping and an additional two curette samples were collected into RNA Lysis Buffer (RLT) (Qiagen) and stored at −80 °C for transcriptome analysis.

### Nasal cell immunophenotyping

Cells were dislodged from curettes and processed immediately. Briefly, the nasal cells were stained for 15 minutes with LIVE/DEAD Fixable Violet Dead Cell Stain (ThermoFisher) before the addition of a cocktail of monoclonal antibodies for 20 minutes at 4 °C protected from light (Supplementary Table 2) as previously described^32^. Samples with less than 500 CD45+ leukocytes or 250 EpCam+ epithelial cells were excluded from analysis. To adjust for the variability of cells collected within and between study participants, absolute cell counts of the target populations were normalized with the absolute number of epithelial cells. The gating strategy is shown in Supplementary Fig. 9.

### Anti-6B CPS IgG

Anti-6B CPS IgG titers in nasal wash samples were measured using a modification of the World Health Organization enzyme-linked immunosorbent assay (ELISA) protocol as previously described^72^. Samples were analyzed in duplicate, and samples with a coefficient of variation of >25% were excluded.

### Nasal fluid multiplex cytokine measurement

Nasal lining fluid was collected using nasosorption filters (Nasosorption, Hunt Developments) and stored at −80 °C. For detection of cytokines in the nasal lining fluid was analyzed as previously described^19^.

### MPO ELISA

Levels of MPO in the nasal wash were determined using the Human MPO DuoSet ELISA Kit (R&D Systems, Abingdon, United Kingdom) following the manufacturer’s instructions as previously described^20^. All samples were run in duplicate with a coefficient of variation <25%.

### Nasal Cell RNA-seq and bioinformatic analysis

#### RNA-seq

Nasal cells were collected in RLT and stored at −80 °C until extraction. RNA extraction (RNeasy; Qiagen), sample integrity assessment (Bioanalyzer; Agilent 2100), library preparation, and RNA-seq (BGISEQ-500RS) were performed at the Beijing Genome Institute.

Quality control of raw sequencing data was done using the FastQC tool. Mapping to a human reference genome assembly (GRCh38) was done using STAR 2.5.0a^73^. Read counts from the resulting binary alignment map files were obtained with featureCounts^74^ using a general transfer format gene annotation from the Ensembl database^75^. The R/Bioconductor package DESeq2 (version 1.34.0) was used to identify differentially expressed genes among the samples after removing absent features (zero counts in more than 75% of samples)^76^. Genes with a *p* value of <0.05 and an absolute fold-change of >2.0 were identified as differentially expressed.

#### Functional analysis

We performed two functional analysis approaches regarding the biological pathways related to aging or carriage status. GSVA was used to identify pathways independent of comparison between groups of samples using R package gsva (version 1.42.0) and log_2_ count per million (CPM) matrix count, and the parameters kcdf = “Gaussian”, min.sz = 15, and max.sz = 500. Gene Set Enrichment Analysis (GSEA) was used to identify which pathways were represented in the ranked transcriptome (log_2_ fold-change), independently of the Differentially Expressed (DE) cut-off^77^ using R package fgsea (version 1.20.0). The criteria used for statistical enrichment for GSEA were a *p* < 0.05 and enrichment of at least five genes for each pathway. The database used for functional analysis was reactome, release 79, and Pathway Browser version 3.7^50^.

#### Co-expression analysis

For co-expression analysis, counts were normalized using log_2_ CPM, and the log_2_ fold-change was calculated for each time point in a subject-wise manner. The co-expression analysis was performed separately for each group (older and younger, using baseline, day 2, and day 9 after pneumococcal challenge and susceptible and protected samples) using the R/Bioconductor package CEMiTool (version 1.18.1)^22^. This package unifies the discovery and the analysis of co-expression gene modules, evaluating whether modules contain genes that are over-represented by specific pathways or altered in a specific sample group. A *p* = 0.05 was applied for filtering genes with low expression levels.

#### M2 CEMiTool module protein-protein interaction network

To identify the relationship between genes (protein-protein interaction) from M2 CEMiTool modules, we filtered which genes were differentially expressed in at least one comparison (susceptible *vs.* protected at baseline, day 2, or day 9 after challenge), and we used the networkanalyst.ca web-based tool (version 3.0). We used the “Gene List Input” option, a list of M2 DE gene symbols, and StringDB^78^ to find the genes interaction evidence under parameters, confidence score cutoff = 900, not required experimental evidence, and zero-order network to use just genes from the input list.

#### Deconvolution analysis

Cell type estimation was performed using the CIBERSORTx web-based tool^79^, with log_2_ CPM gene expression and single-cell atlas data from sorted monocytes cells^80^ and bone marrow and mature neutrophils bulk sequencing^81^ from healthy human donors. Both studies were used as a reference to predict cell type proportion.

### Statistical analysis

To identify the correlation between a pair of features, we first imputed the missing data using the R package missForest (version 1.5) using the default parameters and then calculated the Spearman rho coefficient and *p* values using the R base function cor.test.

Two-tailed statistical tests were used throughout the study. For comparison between study groups, Mann-Whitney test was used. For comparison of study participants within a group over time, Friedmann test with Dunn-Hochberg correction was used. The *p* values were considered significant at <0.05 unless stated otherwise. Data were analyzed using GraphPad Prism (version 9) or R studio (2021.09.2 Build 382) and R (version 4.1.2).

### Study approval

Ethical approval was provided by the East Liverpool National Health Service Research and Ethics Committee (reference numbers 15/NW/0146 and 14/NW/1460) and Human Tissue Authority licensing number 12548.

## DECLARATIONS OF COMPETING INTEREST

The authors have no competing interests to declare.

## FUNDING

This study was funded by the Bill and Melinda Gates Foundation (grant OPP1117728); and the UK Medical Research Council (grant MR/M011569/1). DMF is funded by a NIHR Research Professor grant. HIN is funded by Fundação de Amparo à Pesquisa do Estado de São Paulo (process number: 2018/14933-2).

## CRediT authorship contribution statement

**Britta C. Urban:** Writing – review & editing, Writing – original draft, Supervision, Formal analysis, Data curation. **André N.A. Gonçalves:** Writing – review & editing, Writing – original draft, Methodology, Formal analysis, Data curation. **Dessi Loukov:** Formal analysis. **Fernando M. Passos:** Formal analysis. **Jesús Reiné:** Investigation. **Patrícia Gonzalez-Dias:** Formal analysis. **Carla Solórzano:** Investigation. **Elena Mitsi:** Investigation. **Elissavet Nikolaou:** Investigation. **Daniel O’Connor:** Writing – review & editing. **Andrea M. Collins:** Supervision, Investigation, Data curation. **Hugh Adler:** Investigation, Conceptualization. **Andrew Pollard:** Writing – review & editing. **Jamie Rylance:** Supervision, Conceptualization. **Stephen B. Gordon:** Writing – review & editing, Funding acquisition. **Simon P. Jochems:** Writing – review & editing, Writing – original draft. **Helder I. Nakaya:** Writing – review & editing, Writing – original draft. **Daniela M. Ferreira:** Writing – review & editing, Funding acquisition, Conceptualization.

## Data Availability

The RNA sequencing data have been deposited in the National Center for Biotechnology Information’s Gene Expression Omnibus and are accessible through GEO Series accession number GSE261227. Data underlying graphs are reported in the supporting data file.
