# Supplementary material for: Inflammation of the nasal mucosa is associated with susceptibility to experimental pneumococcal challenge in older adults
Source: Mucosal Immunol. 2024 Oct;17(5):973–89. doi: 10.1016/j.mucimm.2024.06.010 (PMC11464406; doi:10.1016/j.mucimm.2024.06.010)
Supplement: Supplementary Data 1 [file mmc7.pdf]

Supplementary Material

Supplementary Table 1:

	50-64 years						over 65 years					
	-5		2		9		-5		2		9	
Symbol	log2 Fold-change	p-value	log2 Fold-change	p-value	log2 Fold-change	p-value	log2 Fold-change	p-value	log2 Fold-change	p-value	log2 Fold-change	p-value
CXCR1	0.215	7.75E-01	4.822	NA	4.409	6.33E-05	0.524	5.53E-01	1.537	5.68E-02	0.442	6.13E-01
CXCR2	0.210	7.58E-01	4.440	NA	-0.228	7.81E-01	0.537	4.71E-01	1.352	5.73E-02	0.504	5.34E-01
C5AR1	-0.024	9.48E-01	1.804	NA	1.565	4.46E-02	3.239	7.27E-07	1.195	1.57E-02	0.693	2.81E-01
FPR1	0.220	7.37E-01	3.530	NA	2.114	7.17E-02	3.038	1.04E-03	1.572	3.60E-02	0.652	4.15E-01
FPR2	0.340	6.27E-01	4.371	NA	2.039	9.56E-02	2.950	2.02E-03	1.694	2.61E-02	0.724	3.66E-01
ALOX5	-0.009	9.77E-01	1.477	NA	-0.047	9.13E-01	0.241	5.05E-01	0.718	4.20E-02	0.360	4.31E-01
TNFRSF1B	0.076	8.36E-01	2.268	NA	2.165	4.96E-03	2.575	2.27E-04	1.043	6.76E-02	0.498	4.38E-01
ITGAX	-0.277	5.13E-01	1.565	5.90E-02	2.103	1.31E-02	0.441	4.72E-01	1.028	5.50E-02	0.628	3.15E-01
ITGAM	-0.134	7.56E-01	2.854	NA	0.864	2.41E-02	0.665	2.48E-01	0.950	6.12E-02	0.704	2.35E-01
S100A12	0.249	7.09E-01	1.561	1.66E-01	1.192	6.80E-03	3.392	6.03E-05	0.810	2.25E-01	0.833	2.73E-01
DOK3	-0.207	5.89E-01	2.224	NA	-0.177	7.59E-01	0.033	9.53E-01	0.956	7.35E-02	0.112	8.63E-01
LILRB2	0.012	9.81E-01	3.086	NA	2.050	1.12E-02	2.701	1.44E-04	1.194	4.46E-02	0.650	3.17E-01
SELL	0.505	2.07E-01	2.732	NA	1.279	1.79E-03	2.034	8.87E-04	1.360	1.20E-02	0.257	6.71E-01
TYROBP	0.264	4.60E-01	1.661	NA	0.181	7.21E-01	0.245	5.78E-01	0.885	6.85E-02	0.330	5.55E-01
SIGLEC14	0.338	5.83E-01	3.124	NA	1.872	3.64E-02	1.540	7.46E-02	1.017	2.42E-01	0.907	1.38E-01
SIRPB1	-0.121	8.29E-01	3.383	NA	1.047	2.69E-02	2.890	1.06E-04	1.531	5.47E-03	0.747	2.74E-01
PLAUR	-0.235	6.31E-01	1.186	1.35E-01	2.060	6.74E-03	2.975	1.37E-05	1.184	3.11E-02	1.414	2.99E-02
PTPRC	0.070	8.52E-01	2.230	NA	1.647	2.94E-02	2.326	3.45E-04	1.103	3.82E-02	0.663	2.47E-01
TNFAIP6	0.248	7.17E-01	3.148	NA	2.745	2.13E-03	2.824	4.20E-03	1.433	4.30E-02	0.419	5.98E-01
FCGR2A	0.027	9.57E-01	3.598	NA	1.989	5.06E-02	0.566	3.63E-01	1.460	2.18E-02	0.419	5.60E-01
FGR	0.009	9.85E-01	3.406	NA	2.202	1.64E-02	2.491	2.22E-03	1.362	3.53E-02	0.540	4.50E-01
PECAM1	-0.080	8.51E-01	2.211	NA	1.340	5.52E-02	2.324	2.67E-04	0.935	4.95E-02	0.997	8.01E-02
FCGR3B	0.544	4.85E-01	4.852	NA	1.985	1.06E-01	2.901	4.74E-03	1.917	1.46E-02	0.746	4.52E-01
FCAR	0.198	7.70E-01	3.559	NA	2.101	8.66E-02	3.848	4.57E-05	2.206	6.25E-03	1.389	9.76E-02

CCR1	-0.175	7.01E-01	2.370	NA	1.158	4.61E-03	1.863	5.87E-03	0.990	5.82E-02	0.609	3.05E-01
CXCL8	-0.122	8.50E-01	1.331	2.03E-01	2.128	1.49E-02	3.470	2.50E-05	1.664	1.05E-02	1.845	4.04E-03
ICAM1	-0.455	4.39E-01	0.616	3.31E-01	1.663	1.17E-02	1.988	8.93E-04	0.928	2.13E-02	1.092	2.86E-02
SOCS3	-0.264	6.09E-01	2.211	NA	2.047	1.05E-02	2.826	9.82E-05	1.361	2.17E-02	0.542	3.97E-01
FOS	0.471	3.87E-01	2.984	NA	2.278	2.76E-02	3.092	2.42E-05	1.395	1.81E-02	0.754	2.51E-01
IL1R2	-0.028	9.56E-01	2.301	NA	2.308	1.41E-02	2.752	6.26E-04	1.108	9.64E-02	1.197	9.82E-02
IL2RG	-0.147	5.66E-01	1.516	3.58E-03	1.103	7.69E-02	0.195	4.85E-01	0.759	6.60E-02	0.200	6.51E-01
JAK3	-0.085	8.34E-01	1.981	NA	1.913	1.13E-02	0.069	8.81E-01	0.882	8.60E-02	0.192	7.45E-01
IL1B	0.162	8.13E-01	3.712	NA	4.259	4.71E-07	3.035	4.57E-04	1.972	1.33E-02	0.410	6.38E-01
LCP1	0.224	5.35E-01	2.118	NA	1.675	1.61E-02	2.369	1.41E-04	1.111	1.90E-02	0.595	2.74E-01
OSM	0.421	5.48E-01	3.809	NA	3.445	8.40E-03	3.939	2.24E-05	2.126	3.40E-02	0.604	4.97E-01
PTGS2	-0.564	2.93E-01	1.063	1.97E-01	1.143	1.37E-01	2.460	9.92E-05	1.188	3.27E-02	1.189	5.56E-02
CSF3R	-0.052	9.35E-01	4.012	NA	1.503	8.36E-03	0.495	5.30E-01	1.522	2.65E-02	0.599	4.44E-01
HCK	0.108	8.08E-01	2.636	NA	2.088	9.96E-03	0.479	3.87E-01	1.050	7.63E-02	0.491	4.50E-01
FCGR3A	0.273	4.59E-01	1.714	2.24E-02	1.286	4.52E-02	1.803	1.11E-03	0.658	1.83E-01	0.758	7.52E-02
TREML2	-0.449	4.72E-01	3.041	1.49E-03	2.889	8.10E-03	0.712	4.21E-01	1.292	8.54E-02	0.702	3.77E-01
CD300E	0.479	4.64E-01	1.619	NA	4.348	8.85E-06	3.291	2.96E-04	0.592	4.78E-01	1.707	3.32E-02
TREM1	-0.206	7.30E-01	2.700	NA	2.231	4.41E-02	3.471	2.43E-04	1.787	1.70E-02	1.334	9.05E-02
LCP2	0.113	7.62E-01	2.219	NA	2.130	5.61E-03	0.773	1.39E-01	1.029	6.35E-02	0.612	3.08E-01
IFITM2	-0.042	9.15E-01	2.481	NA	1.968	1.12E-02	0.608	2.04E-01	1.152	6.70E-03	0.233	6.89E-01
EGR1	0.615	2.16E-01	2.418	NA	1.636	5.57E-02	2.478	2.73E-04	0.942	8.35E-02	0.540	4.26E-01
GBP5	0.323	3.04E-01	1.363	5.58E-02	1.933	9.09E-03	1.123	9.97E-03	1.230	1.15E-02	0.702	5.25E-02
CXCR4	0.046	8.75E-01	1.380	3.49E-02	1.171	5.49E-02	0.822	1.22E-01	0.987	8.73E-02	0.847	1.07E-01
TLR4	-0.070	8.50E-01	2.137	NA	1.319	7.79E-02	0.592	1.98E-01	1.094	1.44E-02	0.690	1.83E-01

Supplementary Table 2:

Marker	Fluorochrome	Clone	Isotype	Provider	Reference
CD66b	FITC	G10F5	Mouse IgM, k	Biolegend	305104
EpCam	PE	9C4	Mouse IgG2b, κ	Biolegend	324206
TCRVα7.2	PE/Dazzle594	3C10	Mouse IgG1, κ	Biolegend	351730
CD14	PerCPCy5.5	MΦP9	Mouse IgG1, κ	BD Biosciences	562692
HLADR	PECy7	L243	Mouse IgG2a, κ	Biolegend	307616
CD16	APC	3G8	Mouse IgG1, κ	Biolegend	302011
CD3	APC-Cy7	SK7	Mouse IgG1, κ	Biolegend	344818
CD45	Pacific Orange	HI30	Mouse IgG1, κ	ThermoFisher	MHCD4530
Live & Dead	Pacific Blue	NA	NA	Fisher Scientific	L34964
CD4	BV605	RPA-T4	Mouse IgG1, κ	Biolegend	300556
CD107a (LAMP-1)	BV650	H4A3	Mouse IgG1, κ	Biolegend	328638
CD279 (PD-1)	BV711	EH12.2H7	Mouse IgG1, κ	Biolegend	329928
CD8	BV785	SK1	Mouse IgG1, κ	Biolegend	344740

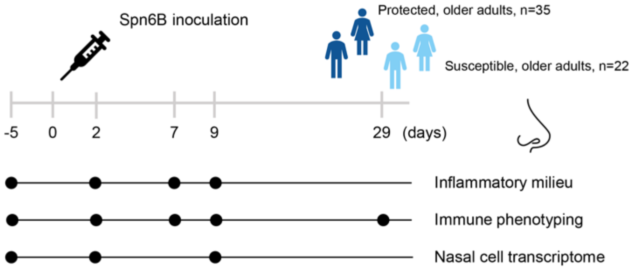

Supplementary Figure 1: Study design. Study design showing inoculation of study participants with Spn6B at day 0 and sample collection before (day -5) and after inoculation with Spn6B (day 2, day 7, day 9, and day 29). Samples collected were nasal lining fluid (nasosorption samples) for determination of the nasal inflammatory milieu and nasal cells (nasal curettage) for immune phenotyping and analysis of the nasal transcriptome. Study participants who remained protected (n=35) are depicted in dark blue, and study participants who became susceptible (n=22) are depicted in light blue.

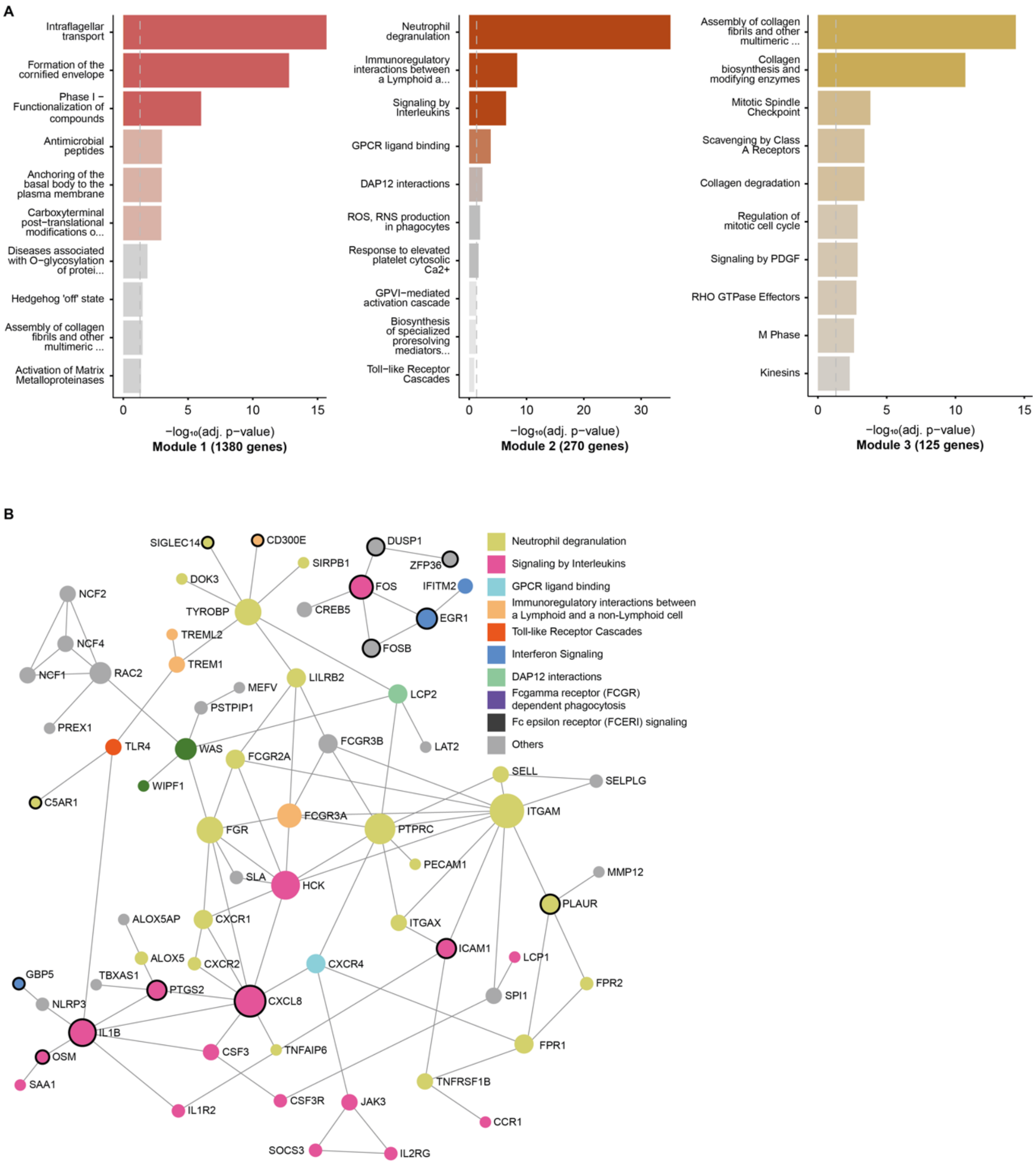

Supplementary Figure 2. Functional analysis of co-expressed gene sets modules. (A) Over-Representation Analysis (ORA) was used to determine biological pathways (using Reactome level 3 gene sets) of co-expressed genes across all modules identified by CEMiTool. (B) Protein-protein interaction network of 216 module 2 genes differentially expressed (susceptible vs protected samples) at at least one timepoint. Genes are colour coded by Reactome pathway and those which were overexpressed at baseline are indicated by a black border. StringDB was used to determine protein-protein interactions using default parameters, a confidence score cut-off of 900 and not required experimental evidence (NetworkAnalysit.ca webtool).

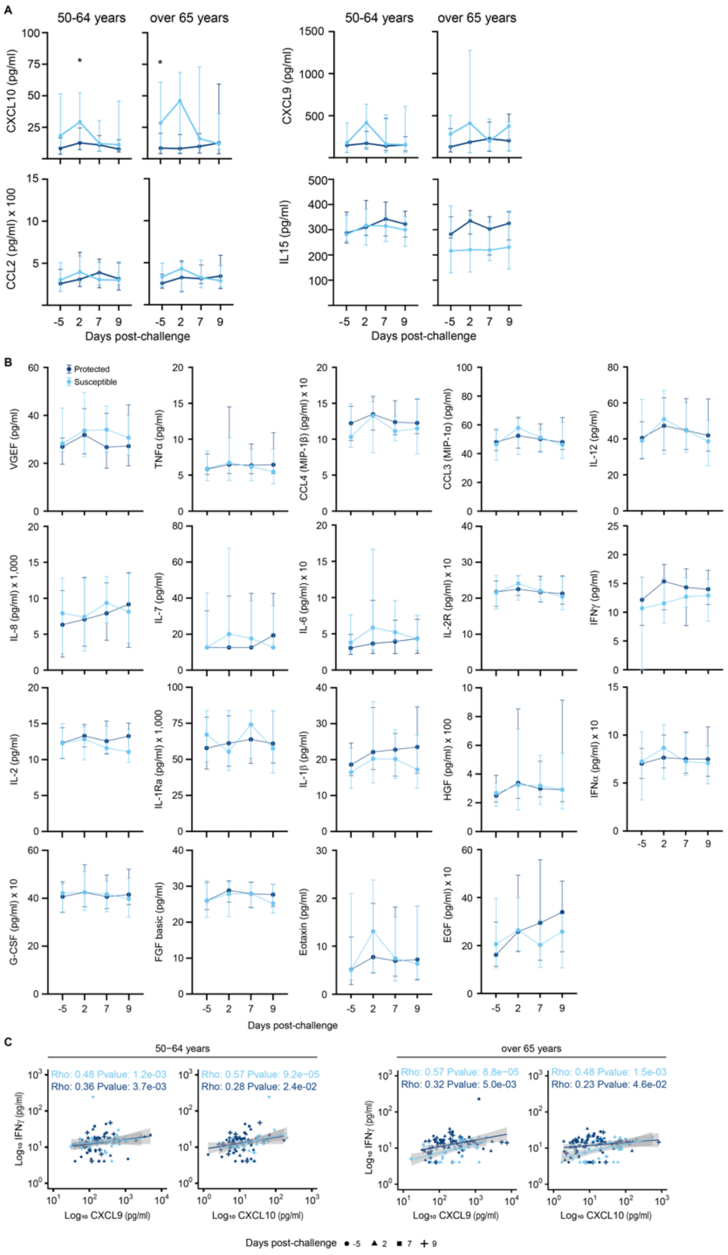

Supplementary Figure 3. Cytokine concentration in the nasal lining fluid of older adults 1 experimentally challenged with S. pneumoniae 6B. (A) Line graphs showing the median and interquartile range of CXCL10, CXCL9, CCL2, and IFNγ concentrations in the nasal lining fluid in older study participants before (day -5) and after (day 2, day 7 and day 9) pneumococcal challenge in susceptible (light blue line, n=22) and protected (dark blue line, n=35) study participants for 50 to 64 years old, and over 65 years old. (B) Line graphs showing the median and interquartile range of cytokine concentrations in the nasal lining fluid in older study participants before (day -5) and after (day 2, day 7 and day 9) pneumococcal challenge in susceptible (light blue line, n=22) and protected (dark blue line, n=35) study participants. (C) Correlation plot of association between IFNγ and CXCL9 or CXCL10

concentrations for 50 to 64 years old, and over 65 years old study participants by carriage status. Correlation was tested using Spearman test.

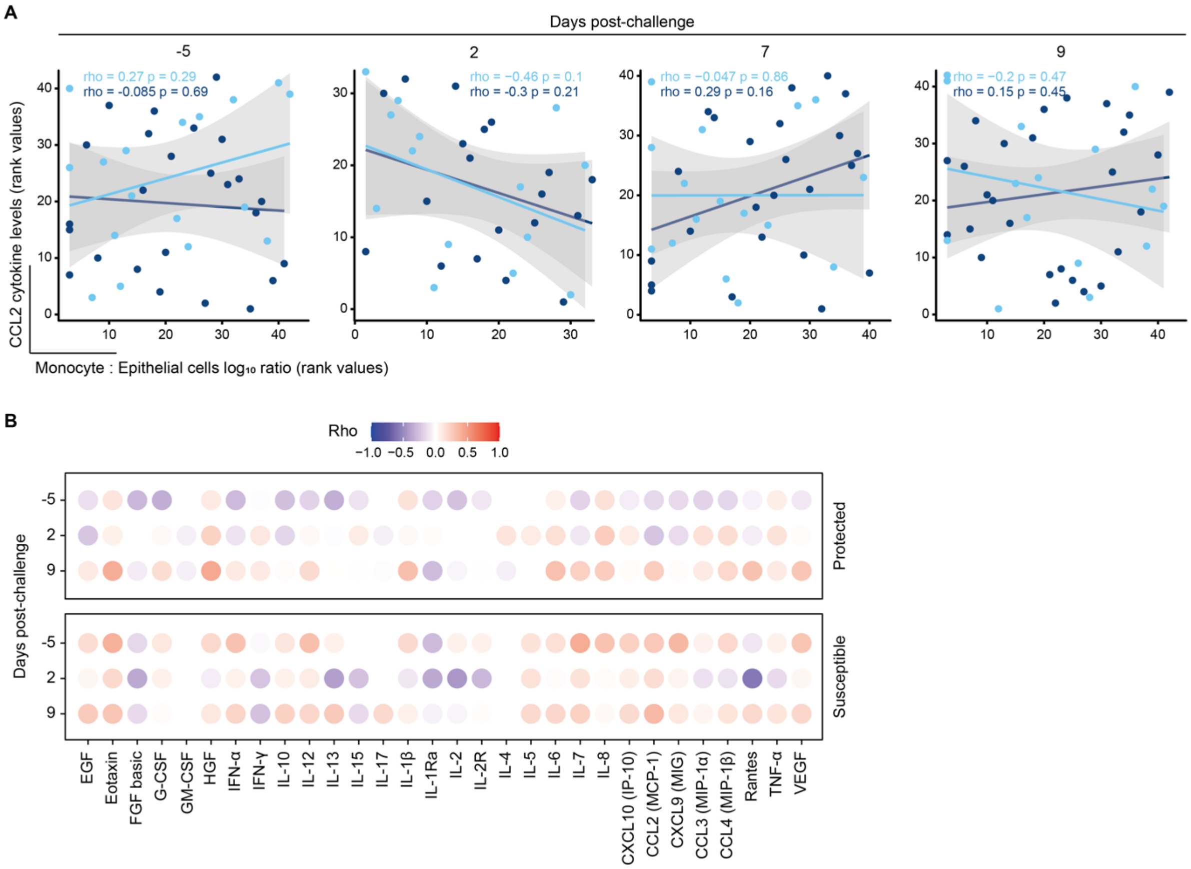

Supplementary Figure 4. Monocytes are not correlated with CCL2 levels in the nasal mucosa before and after pneumococcal challenge. (A) Scatter plot of the CCL2 level (pg/ml) and monocyte: epithelial cells ratio before (day -5) and after challenge (day 2, day 7, and day 9), which showed no correlation in susceptible (light blue dots) and protected (dark blue dots) study participants. (B) Heatmap showing correlation (Spearman rho) between age of protected and susceptible study participants and cytokine concentration before (day -5) and after (day 2 and day 9) pneumococcal challenge. Correlation was tested using Spearman test and Benjamin Hochberg method for adjusting multiple comparison.

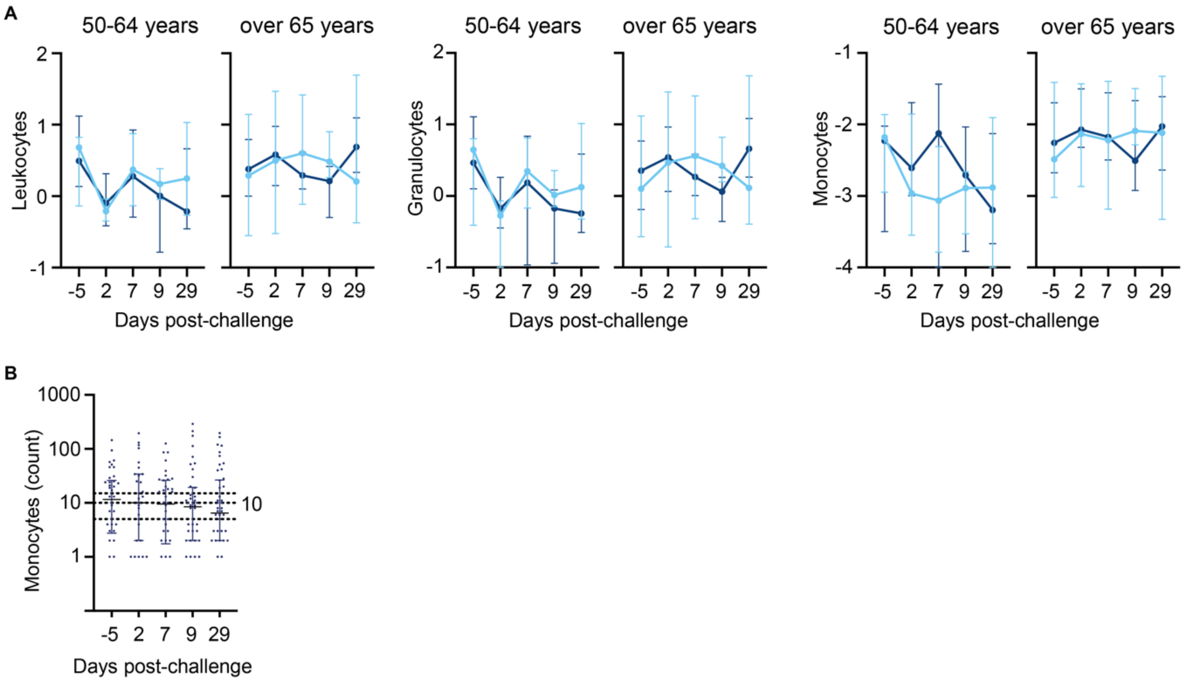

Supplementary Figure 5. Leukocyte subsets cell number in the nasal mucosa of older adults. (A) Line graphs showing the median and interquartile range of leukocytes, granulocytes, and monocytes older study participants before (day -5) and after (day 2, day 7, day 9 and day 29) pneumococcal challenge in susceptible (light blue line, n=22) and protected (dark blue line, n=35) study participants from groups of 50 to 64 years old, and over 65 years old. Cell numbers of each leukocyte subset were normalized to epithelial cells as a ratio to account for differences in the total number of cells obtained for each biopsy. (B) Raw monocyte counts in nasal cell microbiopsies obtained from older study participants across timepoints before (day -5) and after (day 2, day 7, day 9 and day 29) pneumococcal challenge.

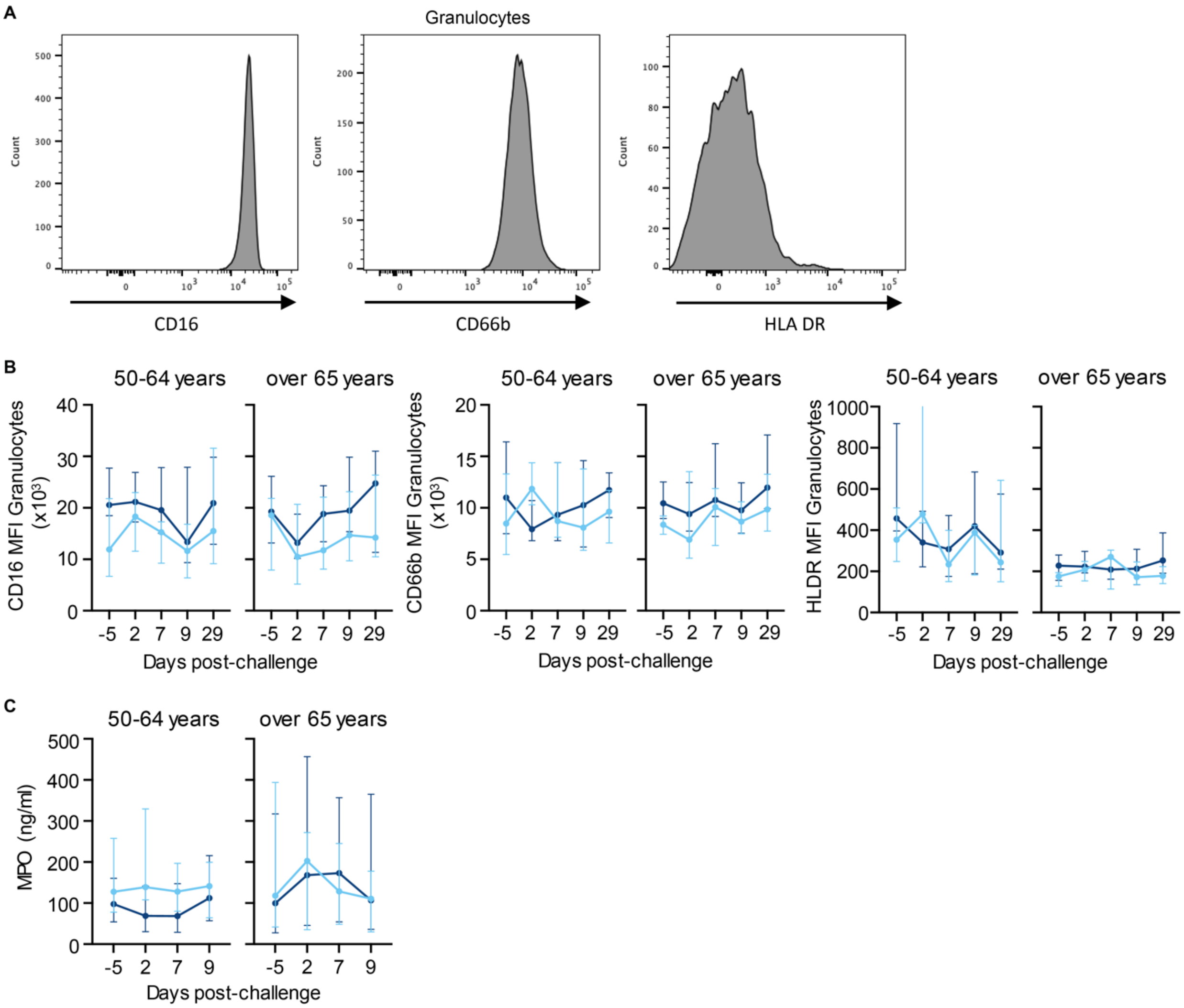

Supplementary Figure 6. Granulocyte subsets cell number in the nasal mucosa of older adults. (A) Histograms showing expression level of CD16, CD66b, and HLADR on granulocytes before pneumococcal challenge from one representative sample. (B) Line graphs showing the median and interquartile range of CD16, CD66b, and HLADR MFI on granulocytes in the nasal mucosa of 50 to 64 years old, and over 65 years study participants old before (day -5) and after (day 2, day 7 and day 9) pneumococcal challenge. (C) At baseline (day -5), the concentration of MPO (Myeloperoxidase) in nasal lining fluid in 50 to 64 years old, and over 65 years old study participants before (day -5) and after (day 2, day 7 and day 9) pneumococcal challenge. Older study participants who remained protected are depicted in dark blue, and study participants who became susceptible are depicted in light blue.

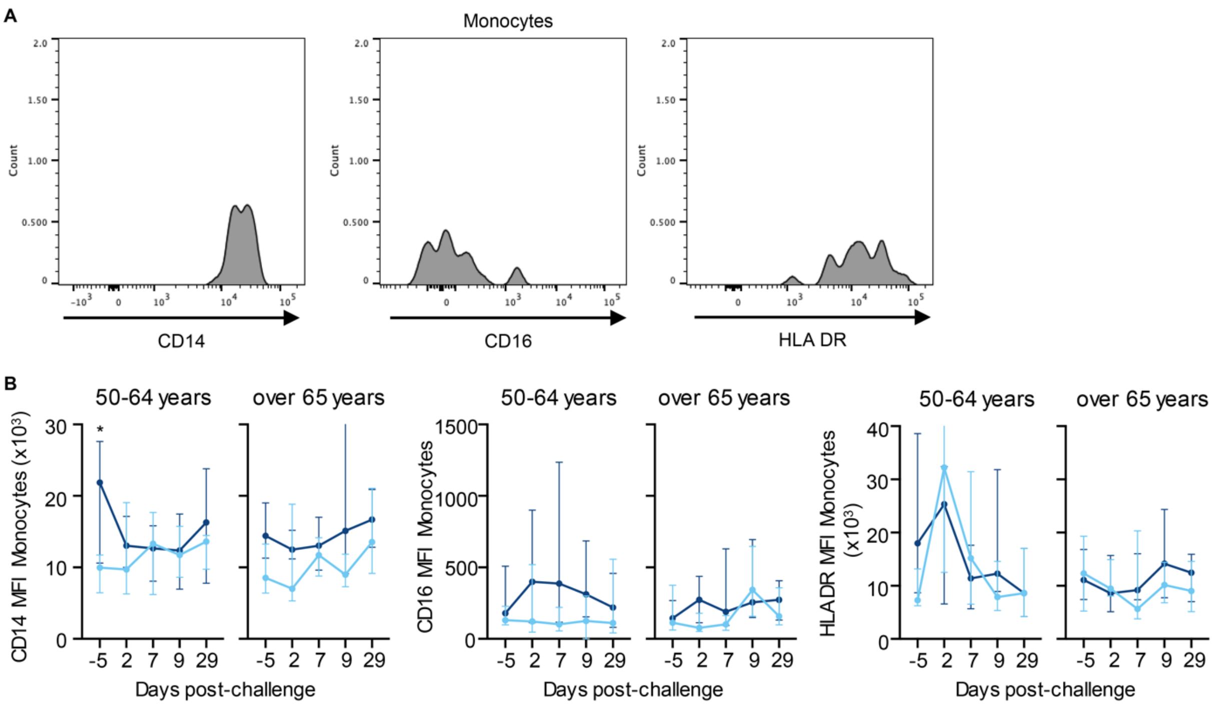

Supplementary Figure 7. Monocyte subsets cell number in the nasal mucosa of older adults. (A) Histograms showing expression level of CD14, CD26, and HLADR on monocytes before pneumococcal challenge from one representative sample. (B) Line graphs showing the median and interquartile range of CD14, CD16, and HLADR MFI on monocytes in the nasal mucosa of 50 to 64 years old, and over 65 years study participants old before (day -5) and after (day 2, day 7 and day 9) pneumococcal challenge. Older study participants who remained protected are depicted in dark blue, and study participants who became susceptible are depicted in light blue.

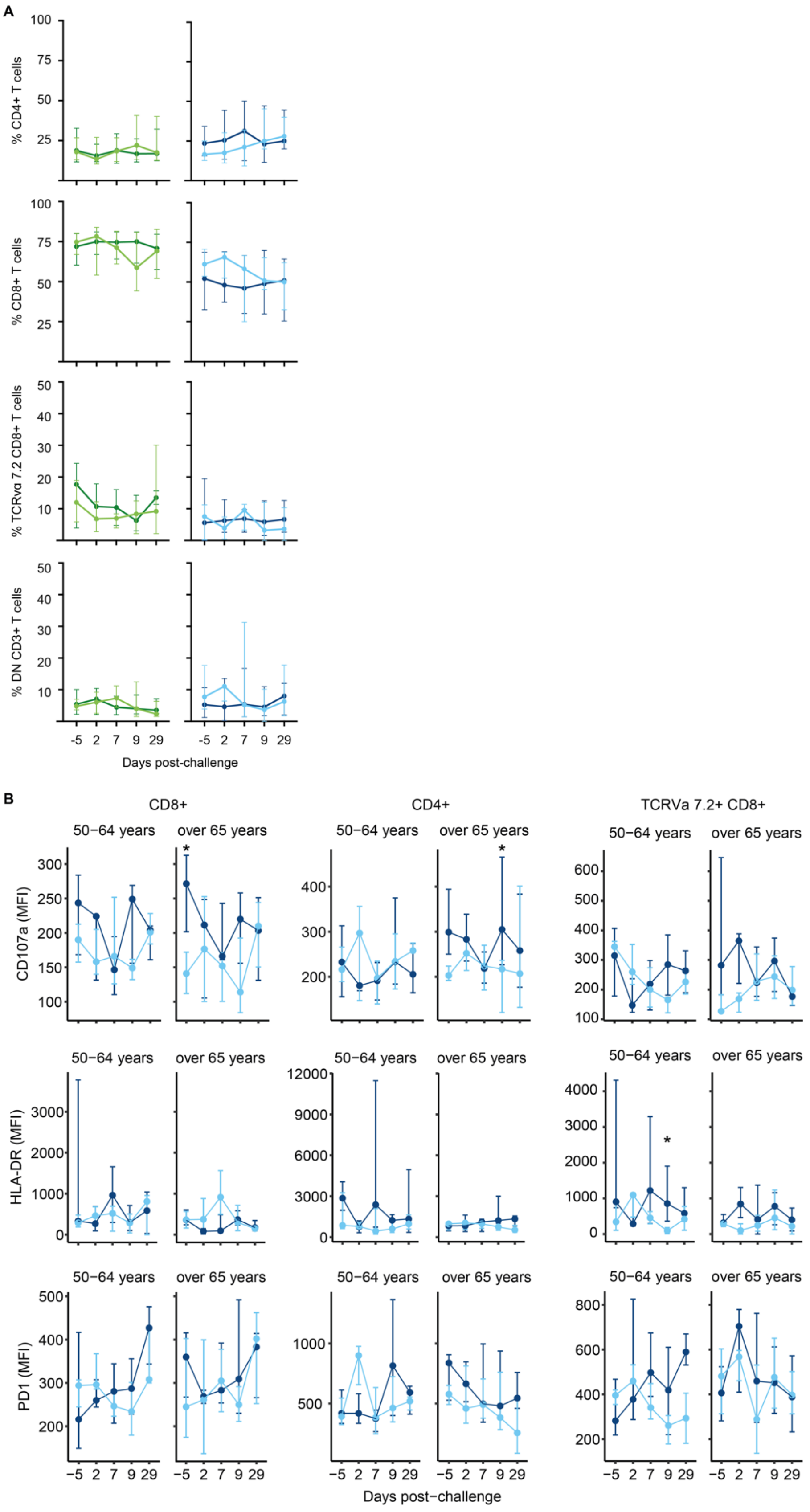
A
% CD4+ T cells
% CD8+ T cells
% TCRvα 7.2 CD8+ T cells
% DN CD3+ T cells
Days post-challenge
B
CD8+
CD4+
TCRVa 7.2+ CD8+
50−64 years
over 65 years
CD107a (MFI)
HLA-DR (MFI)
PD1 (MFI)
Days post−challenge

Supplementary Figure 8. Changes in T cells subsets in younger and older adults before and after pneumococcal challenge. (A) Line graphs showing the median and interquartile range of T cell subsets in the nasal mucosa in younger and older study participants before (day -5) and after (day 2, day 7 and day 9) pneumococcal challenge. Younger study participants who remained protected are depicted in dark green and study participants who were susceptible are shown in light green. (B) Expression levels of CD107a, PD1 and HLA DR on CD8+, CD4+ and TCRVa7.2 CD8+ T cells before (day -5) and after (days 2, 7, 9, 29) challenge with Spn6B in carriage-negative and carriage-positive older study participants for 50 to 64 years old, and over 65 years old study participants. Line graphs show median and interquartile range. Mann Whitney U test *$p<0.05$. Older study participants who remained protected are depicted in dark blue, and study participants who became susceptible are depicted in light blue.

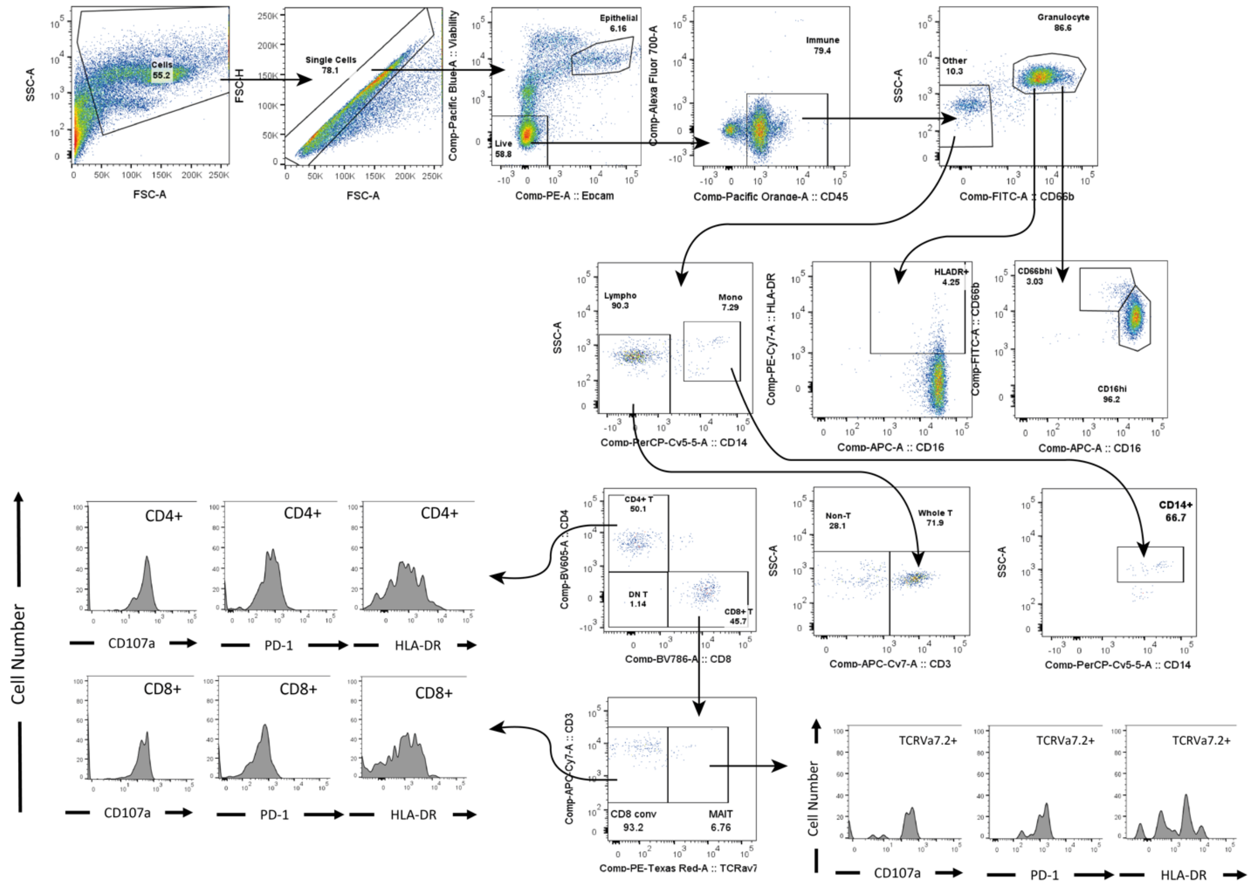

Supplementary Figure 9. Gating strategy for nasal cells of one representative study participant.
